# Disruption of *MIR396e* and *MIR396f* improves rice yield under nitrogen-deficient conditions

**DOI:** 10.1093/nsr/nwz142

**Published:** 2019-09-27

**Authors:** Jinshan Zhang, Zhenyu Zhou, Jinjuan Bai, Xiaoping Tao, Ling Wang, Hui Zhang, Jian-Kang Zhu

**Affiliations:** 1 Shanghai Center for Plant Stress Biology and Center for Excellence in Molecular Plant Sciences, Chinese Academy of Sciences, Shanghai 201602, China; 2 Bellagen Biotechnology Co. Ltd., Ji’nan 250000, China; 3 University of Chinese Academy of Sciences, Beijing 100049, China; 4 School of Biotechnology, Jiangsu University of Science and Technology, Zhenjiang 212018, China; 5 Shanghai Engineering Research Center of Plant Germplasm Resources, College of Life Science, Shanghai Normal University, Shanghai 200234, China; 6 Department of Horticulture and Landscape Architecture, Purdue University, West Lafayette, IN 47907, USA

**Keywords:** microRNA, miR396, *OsGRF4/6/8*, OsGIF1/2/3, nitrogen deficiency, grain yield

## Abstract

The microRNA miR396 directly represses *GROWTH-REGULATING FACTORs* (*OsGRFs*) and has been implicated in regulating rice yield and in nitrogen assimilation. Overexpressing the miR396 targets *OsGRF4* and *OsGRF6* improves rice yield via increased grain size and panicle branching, respectively. Here, we used CRISPR/Cas9 to assess the function of miR396 genes in rice. Knockout of *MIR396ef* (*MIR396e* and *MIR396f*), but not other isoforms, enhanced both grain size and panicle branching, resulting in increased grain yield. Importantly, under nitrogen-deficient conditions, *mir396ef* mutants showed an even higher relative increase in grain yield as well as elevated above-ground biomass. Furthermore, we identified *OsGRF8* as a new target of miR396, in addition to the known targets *OsGRF4* and *OsGRF6*. Disruption of the miR396-targeting site in *OsGRF8* was sufficient to both enlarge grain size and elongate panicles. Our results suggest that rice-seed and panicle development are regulated by miR396ef-GRF4/6/8-GIF1/2/3 modules and that miR396ef are promising targets of genome editing for breeding environmentally friendly rice varieties that require less nitrogen fertilization.

## Introduction

MicroRNAs (miRNAs) are single-stranded, non-coding RNAs of 21–24 nucleotides, which typically load into a silencing complex to direct cleavage or translational inhibition, and thus repression, of complementary messenger RNAs (mRNAs) [1–3]. In plants, miRNAs act as key regulators to control development, growth and stress tolerance [4–6].

Several miRNAs have been shown to control rice yield. For example, miR156 and miR397 affect rice yield by regulating their targets, *OsSPLs* and *OsLACs*, respectively [5–9]. The microRNA miR396 was recently shown to regulate rice-grain yield by targeting the transcription factors growth-regulating factors (GRFs), which execute their function via GRF-interacting factors (GIFs) [10]. miR396 can target and thus limit the expression of both *OsGRF4* and *OsGRF6* in rice [11]. OsGRF4 controls grain development by regulating many brassinosteroid-induced genes and disruption of the miR396 target site in *OsGRF4* enhances grain size and yield in rice [12,13]. OsGRF4 also balances the inhibitory activities of DELLA to promote and integrate nitrogen assimilation, carbon fixation and growth [14], but whether this is regulated by miR396 is not known. OsGRF6 directly promotes the expression of panicle-branching factor genes *OsTAWAWA1* and *OsMADS34*, and decreased expression of *MIR396b* or overexpression of *OsGRF6* increases the panicle-branching number and, in turn, grain yield [15]. Overexpression of the miR396 target genes *AtGRF1, AtGRF2* and *AtGRF5* in *Arabidopsis* leads to the production of larger seeds, indicating that a miR396-GRF regulatory network broadly participates in seed development [16].

miR396 antagonizes the expression of its target *GRF2* in the distal part of leaves, restricting cell proliferation in developed tissues [17]. miR396 also negatively regulates cell proliferation by markedly decreasing the expression of cell-cycle-related genes, such as *CYCLINB1;1* and *TCP4* [17,18]. An miR396-GRF-GIF regulatory network has also been reported to regulate growth, development and abiotic stress tolerance in *Arabidopsis* [19,20]. Overexpression of miR396 in *Arabidopsis* reduces the accumulation of GRF7 [19,21]. GRF7 directly represses *DEHYDRATION-RESPONSIVE ELEMENT BINDING PROTEIN2A* (*DREB2A*) and other genes that are up-regulated in response to abscisic acid; these genes enhance tolerance of dehydration and high-salinity conditions in *Arabidopsis* plants [19].

Given that a single miRNA often targets multiple genes to regulate diverse biological processes, identifying and manipulating key miRNAs is considered a potential strategy to improve agronomic traits of crop plants [22]. Gene-editing technology has been applied to improve agronomic traits and we were interested in using this approach to manipulate key miRNAs and generate resources for breeding elite crop varieties. Here, we studied the individual functions of the eight members of the miR396 gene family in rice. We discovered that two members, miR396e and miR396f (miR396ef), regulate seed and panicle development. *mir396ef* mutants showed increases in grain size and panicle branching, which resulted in increased grain yield. Importantly, *mir396ef* mutations showed a more substantial increase in grain yield and above-ground biomass under conditions of nitrogen deficiency. We demonstrated that *OsGRF4*, *OsGRF6* and *OsGRF8* are the main targets of miR396ef and that miR396-GRF4/6/8-GIF1/2/3 modules regulate rice-grain and panicle development. Our work suggests that miR396ef is an attractive target for generating high-yield rice plants that are less dependent on nitrogen fertilization.

## Results

### miR396ef isoforms regulate grain development in rice

**Figure 1.**
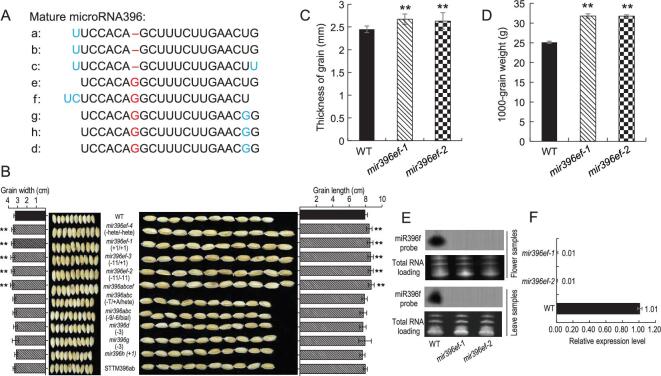
miR396ef isoforms regulate rice-grain development (A) miR396 members in rice. Isoform differences are highlighted in blue. Red color indicates the difference at position seven. (B) Comparison of the 10-grain width and 10-grain length between mir396 mutant plants and wild-type (WT). The genotypes are labeled after the name of mutants in the brackets. Plus (+) and minus (−) and subsequent number represent the nucleotides inserted and deleted at the sgRNA target sites, and mutations on different mir396 members are separated by slashes. The ‘hete’ means heterozygous mutation on the corresponding members. The ‘bial’ means biallelic mutation on the corresponding member. (C) The grain thickness of *mir396ef* and WT plants. (D) The 1000-grain weight of *mir396ef* and WT plants. (E) Northern blotting detection of miR396ef in flowers and leaf samples of WT and *mir396ef* plants. Total RNA stained with ethidium bromide was used as a loading control. (F) Stem-loop RT-qPCR of miR396ef in WT and *mir396ef* leaf samples. U6 was used to normalize samples. Data are presented as means ± SD (*n* = 100 in (B)–(D); *n* = 3 in (F)). *P*-values (versus the WT) were calculated with Student’s *t*-test, two-tailed. **P < 0.01.

Rice contains five different miR396 isoforms, encoded by eight genes (Fig. 1A). According to the plant miRNA database PmiREN (Plant miRNA ENcyclopedia, http://www.pmiren.com/), the family members *MIR396e* and *f* have the highest expression in reproductive tissues, such as inflorescence, florets and spikelets (Supplementary Table 1) [23,24]. *MIR396c* is also expressed in these reproductive tissues, but at a relatively lower level (Supplementary Table 1) [23,24]. To identify the miR396 isoforms that modulate rice-seed and panicle development, we used CRISPR-Cas9 and five sgRNA constructs to knock out the eight *MIR396* members (Supplementary Fig. 1). miR396a–c and miR396e–f have the same mature sequences, respectively, and each group was targeted by one construct. Although miR396 d, g and h also have the same mature sequence, they are located on the complementary strands of different genes, and therefore were targeted by three different constructs to avoid functional disruptions of overlapping genes (Supplementary Fig. 1D).

CRISPR lines with mutations in mature miR396 regions, which should disrupt function, were selected for further study. Unexpectedly, only *mir396ef* mutants exhibited grain-size phenotypes, whereas the remaining *mir396* mutants displayed no differences compared with wild-type (WT) (Fig. 1B and Supplementary Figs 2 and 3). In addition, *mir396abc* triple mutants*, mir396d* and *mir396g* single mutants (with non-frameshift mutations in overlapping genes) and *mir396h* mutants exhibited no differences in morphological phenotypes compared to WT, including plant height (Supplementary Figs 2A and 3A, E and I), grain length, grain width (Fig. 1B and Supplementary Figs 2B and C and 3B, C, F, G, J and K) and panicle architecture (Supplementary Figs 2D and E and 3M and N). We also applied the short tandem target MIMICs (STTM) technology to block miR396ab functions (Supplementary Fig. 4A and E). The STTM-miR396 lines showed similar seed length, seed width and panicle branching compared with WT (Supplementary Fig. 4B–D).

In contrast, compared with the WT, *mir396ef* mutants showed obvious increases in grain length, width and thickness (increased 8.46%, 7.95% and 8.16%, respectively) (Fig. 1B and C). The 1000-grain weight was also significantly increased in *mir396ef* (31.78 ± 0.55 g) compared to WT (25.12 ± 0.30 g) (Fig. 1D). We obtained many *mir396ef* double-mutant CRISPR lines with different genotypes, all of which displayed the same seed phenotypes, confirming the functions of *MIR396ef* during seed development (Fig. 1B and Supplementary Fig. 5A and B). We generated *mir396abcef* mutants by crossing *mir396abc* with *mir396ef* mutants, which showed similar grain length and width as *mir396ef* (Fig. 1B). The mature isoform of miR396ef was almost undetectable in *mir396ef* leaf and flower (Fig. 1E and F), indicating successful elimination of *MIR396e* and *f*.

Due to the extremely high genome-editing efficiency at the mi396e and f target sites, we obtained only *mir396ef* double mutants. To obtain an *mir396e* single mutant, we designed an sgRNA to specifically disrupt *MIR396e* (Supplementary Fig. 6A and B). The grain length and width of *mir396e* mutants are 4.77% and 5.58% larger than the WT, respectively (Supplementary Fig. 6C and D). The seed phenotypes of *mir396e* are weaker than that of *mir396ef* double mutants (Supplementary Fig. 6C), suggesting that both *MIR396e* and *MIR396f* contribute to the grain-size development. We examined putative off-target loci for miR396abc and miR396ef target sequences and did not observe any mutations (Supplementary Fig. 7). These results indicate that both miR396e and f isoforms are involved in rice-grain development, although *mir396f* single mutants are needed to confirm the function of miR396f.

### *mir396ef* mutants display simultaneous increases in grain size and panicle branching

**Figure 2.**
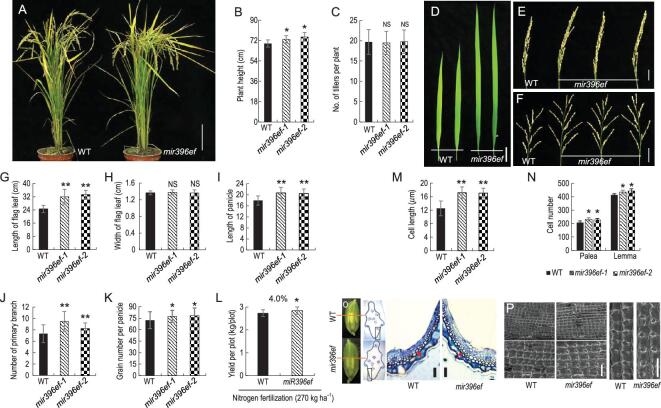
Morphological and growth phenotypes of *mir396ef* plants. (A) Gross morphologies of WT and *mir396ef* plants at maturity. Scale bar, 10 cm. (B) Plant height of WT and *mir396ef*. (C) Tiller number per plant of WT and *mir396ef*. Leaf (D), panicle (E) and panicle-branching (F) phenotypes of WT and *mir396ef* plants. Scale bars: 50 mm in (D); 30 mm in (E) and (F). Flag-leaf blade length (G), flag-leaf blade width (H), panicle length (I), number of primary branchings per panicle (J) and grain number per panicle (K) of WT and *mir396ef* plants. (L) Grain yield per plot of WT and *mir396ef* with nitrogen fertilization. One hundred plants (10 × 10) were cultivated in 2.0 × 2.0 (m) plots. (M) Cell length of grain spikelet hulls in WT and *mir396ef* plants. (N) Cell number of spikelet hull palea and lemma in WT and *mir396ef* plants. (O) Cross-sections of spikelet hulls of WT and *mir396ef* plants, and magnified view of the boxed cross-section area. The red line indicates the position of the cross-section. The rectangle indicates the position of the magnification of the hull. The red arrow points to the palea cells. Scale bars, 50 μm. (P) Scanning election microscopy images of the outer surface of glume in WT and *mir396ef*. Scale bars, 100 μm. Data are presented as means ± SD (*n* = 20 in (B), (C), (G)–(K) and (M); *n* = 5 in (L) and (N)). *P*-values (versus the WT) were calculated with Student’s *t*-test, two-tailed. *P < 0.05; **P < 0.01. NS, not significant.

The increased seed size of the *mir396ef* mutants was associated with a significantly enlarged grain husk, as demonstrated by both histological sectioning and scanning electron microscopy (Fig. 2O and P). The length and number of inner palea and lemma cells were significantly increased in the *mir396ef* grain husk compared with the WT (Fig. 2M and N). An *MIR396e*-promoter::GUS assay indicated that *MIR396e* was expressed in grain hull, spikelet, leaf and root (Supplementary Fig. 8), consistently with its regulation of grain-husk development.

We found that *mir396ef* mutant plants (74.13 ±3.54 cm) were taller than the WT (69.33 ± 3.20 cm), though the tiller number was similar to WT (Fig. 2A–C). In addition, compared to the WT flag leaf, the *mir396ef* flag leaf was 32.8% longer but had the same width (Fig. 2D, G and H), resulting in an increased leaf area. The photosynthetic rates of the flag leaf showed no difference between the *mir396ef* and the WT in the paddy field (Supplementary Fig. 9). The unchanged photosynthetic rates with increased leaf area would enhance overall photosynthesis, perhaps leading to the seed increase in *mir396ef* mutants relative to WT.

Rice grains grow on the panicle with numerous branches. Longer panicles and more panicle branches generally correlate with more grains. Compared to WT, *mir396ef* lines had longer panicles (20.55 ± 1.83 vs 17.93 ± 1.64 cm), more primary branches (8.8 ± 1.34 vs 7.33 ± 1.56) and higher grain numbers per panicle (77.82 ± 8.89 vs 72.35 ± 10.96) (Fig. 2E, F, I, J and K). However, the *mir396ef* panicles were partly enwrapped by the flag leaf, which affects the fertility and filling of the enwrapped panicle (Supplementary Fig. 10A–C).

The phenotypes of *mir396ef*, including a longer flag leaf for enhanced photosynthesis, larger grain size for more grain weight and more grains per panicle, suggest that *mir396ef* could be valuable targets to increase grain yield. Our paddy-field plot-yield test revealed an approximately 4% increase in grain yield on conventional agricultural 270 kg ha^−1^ nitrogen fertilizer (Fig. 2L). Like WT mature pollens, mir396ef pollens were deeply stained by iodine–potassium iodide (I_2_-KI) and their fertility was not changed (Supplementary Fig. 10D–F). The yield increase of *396ef* was below what we expected, perhaps due to a higher rate of empty grains in the *mir396ef* mutants than in WT (Supplementary Fig. 10C).

We also tested grain-quality traits in the *mir396ef* mutants. The brown and polished grains of *mir396ef* mutants had a more ‘white splotch’ appearance compared with the WT-grain appearance (Supplementary Fig. 11A and B). The alkali-spreading value (gelatinization temperature) as well as the percentage and degree of chalkiness were increased (Supplementary Fig. 11C, D and F), whereas the gel consistency was decreased in the *mir396ef* mutants compared with the WT (Supplementary Fig. 11E).

### *mir396ef* mutants exhibit significantly increased grain yield under nitrogen-deficient conditions

The miR396 target *OsGRF4* confers increased nitrogen assimilation and biomass accumulation in rice [14]. To investigate the response of miR396 to nitrogen treatments, we examined the expression of miR396 family members in seedlings grown in hydroponic culture either with or without nitrogen supply. We found that the levels of miR396f increased transiently in seedlings grown under nitrogen-deficient conditions, reaching a maximum at about 6 hours after exposure to nitrogen deficiency, then decreasing and returning to normal levels at about 24 hours (Supplementary Fig. 12). In contrast, miR396e expression did not change in response to nitrogen deficiency (Supplementary Fig. 12).

**Figure 3.**
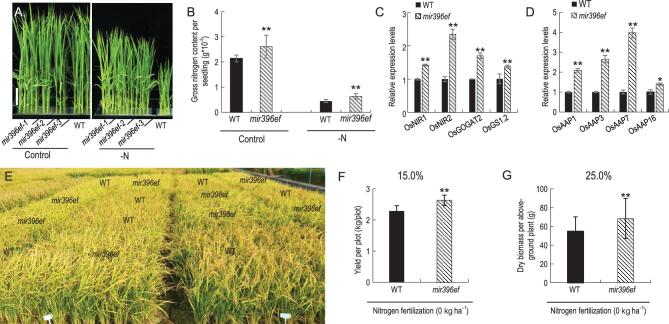
Grain yield of WT and *mir396ef* in a nitrogen-deficient environment. (A) Nitrogen-deficiency response assay in WT and *mir396ef* plants. Scale bar, 5 cm. (B) The gross nitrogen content per seedling with and without nitrogen supplied in WT and *mir396ef* plants. (C) and (D) The expression level of nitrogen-related genes in WT and *mir396ef* seedling samples detected by RT-qPCR. ACTIN1 was used to normalize samples; relative expression levels were measured using the 2^–ΔΔCt^ analysis method. (E) Plots yield test of WT and *mir396ef* in nitrogen-deficient paddy fields. (F) Grain yield per plot of the WT and *mir396ef* without nitrogen fertilization. One hundred plants (10 × 10) were cultivated in 2.0 × 2.0 (M) plots. (G) Dry biomass of the above-ground per WT and *mir396ef* plants in nitrogen-deficient paddy fields. Data are presented as means ± SD (*n* = 3 in (B)–(D); *n* = 5 in (E)–(G)). *P*-values (versus the WT) were calculated with Student’s *t*-test, two-tailed. *P < 0.05; **P < 0.01.

Next, we examined the nitrogen assimilation of the *mir396ef* mutants at the rice-seedling stage. We found that seedlings from *mir396ef* plants had a significantly higher nitrogen content than those from WT plants, when grown in hydroponic culture either with or without nitrogen supply (Fig. 3A and B). Recently, Li *et al.* showed that OsGRF4 recognizes the GCGG core motif by ChIP-Seq; ChIP-PCR and Electrophoretic Mobility Shift Assay (EMSA) confirmed that OsGRF4 associates with promoters that contain this motif and regulate genes involved in ammonia and nitrate metabolism, including *GOGAT2*, *NIR1* and *GS1.2*, etc. [14]. To determine whether genes involved in nitrogen assimilation and utilization are differentially expressed in *mir396ef* mutant and WT seedlings, we performed reverse-transcription quantitative PCR (RT-qPCR). Specifically, we examined the expression of *NIR1*, *NIR2*, *GOGAT2* and *GS1.2*, which encode enzymes involved in nitrogen assimilation [14]. We also examined the *AMINO ACID PERMEASE* genes (*OsAAPs*), which are nitrogen-use-efficiency-related genes that determine amino-acid delivery from source to sink in plants [25,26]. All of these genes we examined showed increased expression in *mir396ef* mutant seedlings compared to WT seedlings (Fig. 3C and D). These results suggest that *MIR396ef* is involved in nitrogen assimilation and utilization in rice.

Next, we determined whether the improved nitrogen assimilation and utilization of *mir396ef* mutant plants affect growth and yield under nitrogen-deficient conditions. We found that *mir396ef* mutants grew substantially taller than WT plants when grown in hydroponic culture without nitrogen supply (Fig. 3A). Moreover, *mir396ef* plants showed a substantial yield increase (∼15%) compared with the WT under nitrogen-deficient conditions (0 kg ha^−1^ nitrogen fertilizer application) in the paddy field (Fig. 3E and F). The yield of *mir396ef* under low-nitrogen conditions was comparable to the yield of WT under normal nitrogen-fertilization conditions (Supplementary Table 2). Interestingly, *mir396ef* showed a 25% increase in above-ground dry biomass per plant under nitrogen-deficient conditions compared to WT plants (Fig. 3G).

### Regulation of grain size and branch length by an miR396ef-GRFs-GIFs module

**Figure 4.**
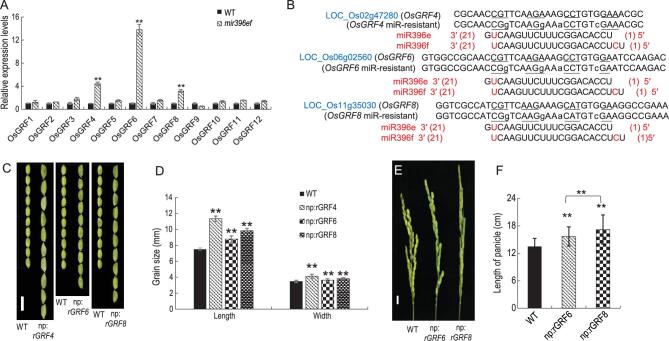
miR396 regulates grain size and panicle length via *OsGRF4*, *OsGRF6* and *OsGRF8*. (A) The expression profiles of 12 *OsGRFs* genes in WT and *mir396ef* leaf samples. (B) The mutations introduced at the miR396 binding site of *OsGRF4*, *OsGRF6* and *OsGRF8* mRNAs to generate miR396-resistant *OsGRF4*, *OsGRF6* and *OsGRF8* driven by their respective native promoters (namely *np:rOsGRF4*, *np:rOsGRF6* and *np:rOsGRF8*). (C) The grain-length phenotypes of *np:rOsGRF4*, *np:rOsGRF6* and *np:rOsGRF8*. Scale bar, 10 mm. (D) Comparison of the grain width and grain length between *np:rOsGRF4*, *np:rOsGRF6* and *np:rOsGRF8* with WT, respectively. (E) Panicle length of *np:rOsGRF6 and np:rOsGRF8*. Scale bar, 15 mm. (F) Comparison of panicle length between *np:rOsGRF6* and *np:rOsGRF8* with WT. Data are presented as means ± SD (*n* = 20 in (D); *n* = 5 in (F)). *P*-values (versus the WT) were calculated with Student’s *t*-test, two-tailed. **P < 0.01.

Twelve GRF transcription factors in rice harbor the miR396 target site [27]. We found that only *OsGRF4*, *OsGRF6* and *OsGRF8* were up-regulated in *mir396ef* plants compared to WT (Fig. 4A). RLM-RACE (5^′^ RNA ligase mediated rapid amplification of cDNA ends) analysis showed that miR396 could direct the cleavage of *OsGRF4* and *OsGRF6* mRNAs *in vivo* at a specific site within the miR396 pairing region with leaf samples (Supplementary Fig. 13A). Interestingly, in leaf samples, miR396 caused the cleavage of *OsGRF8* mRNA at a site 118 bp downstream of the miR396 pairing region, and results of transient co-expression assays in *Nicotiana benthamiana* leaves are consistent with such a cleavage (Supplementary Figs 13A and 14). However, in flower samples, miR396 could direct the cleavage of *OsGRF8* mRNA at the predicted miR396 pairing region (Supplementary Fig. 13A).

To investigate whether miR396-mediated regulation of *OsGRF4*, *OsGRF6* and *OsGRF8* control seed and panicle development, we constructed miR396 target-resistant versions of *OsGRF4*, *OsGRF6* and *OsGRF8* (designated np:*rGRF4*, np:*rGRF6* and np:*rGRF8*, respectively) (Fig. 4B) and introduced them into rice under the control of their respective native promoters. We found that np:*rGRF4*, np:*rGRF6* and np:*rGRF8* lines exhibited larger grain sizes than WT plants (Fig. 4C and Supplementary Fig. 13B–D), with np:*rGRF4* showing the largest increase and np:*rGRF6* showing the smallest increase (Fig. 4D and Supplementary Fig. 13B–D). Both np:*rGRF6* and np:*rGRF8* lines showed increased panicle lengths (Fig. 4E and F and Supplementary Fig. 13I), but their branching numbers remained unchanged compared to WT. The np:*rGRF4* lines displayed similar panicle length and branching numbers to WT (Supplementary Fig. 13H). These results suggest that the miR396ef members regulate seed and panicle development by regulating their target genes *OsGRF4*, *OsGRF6* and *OsGRF8*.

**Figure 5.**
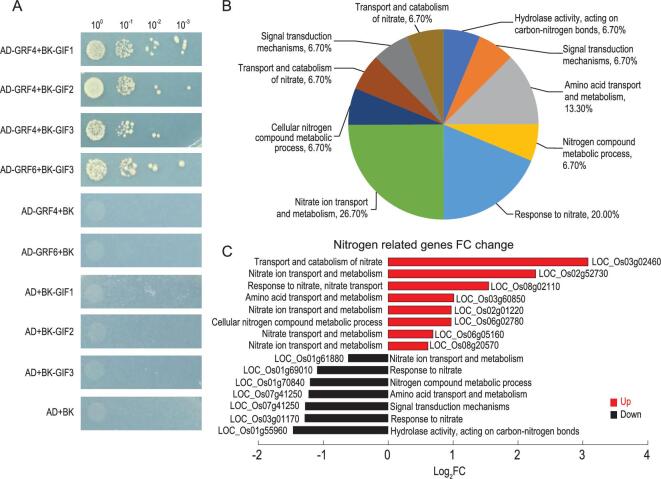
The interactions between OsGIFs and OsGRFs, and differentially expressed genes in *mir396ef* plants. (A) OsGRF4 and OsGRF6 interact with OsGIF1/2/3 in a yeast two-hybrid assay. (B) Nitrogen-related genes with differential expression in *mir396ef* versus WT leaf. (C) The expression of nitrogen-related genes in *mir396ef* compared with WT leaf. FC, fold change (mir396ef/WT).

GRFs have been shown to interact with transcription coactivators GIFs [12,20]. To uncover GIFs that interact with OsGRF4 and OsGRF6, we performed a yeast two-hybrid assay. We found that OsGIF1, OsGIF2 and OsGIF3 interacted with OsGRF4, and OsGIF3 also interacted with OsGRF6 (Fig. 5A). Biomolecular fluorescence complementation (BiFC) assays confirmed these interactions in tobacco leaves (Supplementary Fig. 15). The expression of *OsGIF1* was significantly increased in mir396ef plants (Supplementary Fig. 16A). We used the CRISPR/Cas9 technology to generate *gif1* mutants and observed the expected short-leaf phenotype (Supplementary Fig. 16B). *osgif1* mutants also exhibited smaller plant stature and aborted seed phenotypes compared to WT (Supplementary Fig. 16B and C). These findings suggest that rice-seed and panicle development are regulated by the miR396ef-GRF4/6/8-GIF1/2/3 module.

To further explore the function of miR396ef in plants, we used RNA-sequencing analysis to examine the transcriptomes of leaves and young spikelet tissues from *mir396ef* and WT plants. We detected 558 DEGs (differentially expressed genes, ratio > 1.5) in young spikelet tissues, but only 189 DEGs in leaves. We observed 314 and 127 up-regulated genes in *mir396ef* mutant spikelet and leaves, respectively, compared to WT. In addition, 244 and 62 genes were down-regulated in *mir396ef* mutant spikelet and leaves compared to WT, respectively (Supplementary Fig. 17A). We observed 15 DEGs encoding ‘nitrogen-related genes’ in leaves and enrichment analysis of the DEGs identified ‘nitrate ion transport and metabolism’ as the top annotated category (Fig. 5B and C). In addition, 26 DEGs were related to brassinonsteroid-induced genes and 21 DEGs were related to auxin signaling, consistently with auxin- and brassinosteroid-mediated regulation of spikelet number and grain size (Supplementary Fig. 18A and B) [12,15]. We confirmed the expression of key genes within brassinosteroid and auxin-signaling pathways by RT-qPCR (Supplementary Fig. 19A and B). We also observed increased expression of the cell-proliferation-related genes, two *CYCLINB1;1* homologous genes and one *TCP4* homologous gene, in *mir396ef* mutant, consistently with the increases in cell number (Supplementary Fig. 19C–E).

## Discussion

Here, we investigated the role of the miR396 gene family in controlling grain yield in rice, with the goal of identifying targets and strategies for breeding elite crop varieties. The miR396 target gene *OsGRF4* increases grain size by activating brassinosteroid responses [12], whereas the miR396 target gene *OsGRF6* promotes panicle branching through the activation of several auxin biosynthesis factors [15]. Thus, targeting miR396 itself would be expected to enhance both grain size and panicle branching. We examined the miR396 members by CRISPR/Cas9 editing (Fig. 1A and Supplementary Fig. 1) and found that *mir396ef* mutants showed the expected traits of increased grain size and panicle branching. It was reported that miR396b negatively regulates panicle branching by targeting *OsGRF6*, and STTM-miR396b plants showed increased panicle branching and grain yield [15]. However, in our data, STTM-miR396ab lines showed unchanged panicle branching compared with the WT. This discrepancy may be caused by the different genetic backgrounds, since the reported STTM-miR396b lines were in the *indica* background whereas our STTM-miR396ab lines are in the *japonica* background. More recently, Miao *et al.* [28] reported that *mir396e* single mutant rice had a similar grain size to the WT Xiushui 134. However, in our data, the grain length and width of *mir396e* mutants are larger than the WT *Nipponbare*, which may also be explained by the background difference.

We found that *mir396ef* mutants have several beneficial traits relative to WT plants, including increased plant height (Fig. 2A and B), enhanced length of the flag leaves and panicles (Fig. 2D and E), increased primary branch and grain number in panicle (Fig. 2F, J and K) and increased grain size and weight (Fig. 1B–D). Furthermore, the cell length and cell number in the grain hull of *mir396ef* mutants are both increased, which enlarged the sink size. Just as in *OsGRF4*-overexpression grains, the *mir396ef* mutant showed decreased grain quality because of increased chalkiness (Supplementary Fig. 11), which could be offset by alleles of high-quality genes [29].

miR396 attenuates cell proliferation in developing leaves and negatively regulates leaf growth in *Arabidopsis thaliana* [17,27]. We found that *mir396ef* mutant rice plants also showed an obvious increase in leaf length. In addition, the np:*rGRF4*, np:*rGRF6* and np:*rGRF8* lines all exhibited increased leaf length and enwrapped panicles (Supplementary Fig. 13E–G). The *np:rGRF4* plants are obviously shorter than the WT, perhaps because the *np:rGRF4* panicles were severely enwrapped by the flag leaf (Supplementary Fig. 13E). The significant increases in the source leaves of *mir396ef* mutants could explain the increased grain yield and biomass. Although the grain yield of *mir396ef* mutants was only 4.0% greater than that of WT in test plots with normal nitrogen fertilization (Fig. 2L), it was 15.0% greater in natural paddy fields without nitrogen fertilization (Fig. 3E and F). Further, the above-ground biomass of *mir396ef* mutant plants was 25.0% more than that of WT plants under nitrogen-deficient conditions (Fig. 3G). These results suggest that *mir396ef* mutations cause enhanced nitrogen utilization, consistently with increased *OsGRF4* function, which has been shown to balance the inhibitory activities of DELLA to promote and integrate nitrogen assimilation, carbon fixation and growth (Fig. 3C and D) [14]. This notion is supported by our discovery that nitrogen transport and metabolism genes are up-regulated in *mir396ef* mutants (Fig. 5B and C).

*OsGRF4* and *OsGRF6* have been reported as targets of miR396ef. Based on expression and mRNA cleavage studies, we uncovered *OsGRF4*, *OsGRF6* and *OsGRF8* as major targets of miR396ef (Fig. 4A and Supplementary 13A). Plants expressing miR396ef-resistant versions of target genes, np:*rGRF4*, np:*rGRF6* and np:*rGRF8*, all exhibited larger grain size to varying degrees, with the largest increase in np:*rGRF4*. However, the panicle length and branching numbers of np:*rGRF4* were unchanged compared to WT (Fig. 4C and D and Supplementary Fig. 13E). Panicle lengths were increased in np:*rGRF6* and np:*rGRF8* (Fig. 4E and F), but branching numbers were unaffected (Supplementary Fig. 13H and I). In summary, we did not observe synchronous increases in grain size and panicle branching in np:*rGRF4*, np:*rGRF6* and np:*rGRF8* plants. These data suggest that the seed-size and panicle-length improvement of *mir396ef* mutants is due to the combined functions of OsGRF4, OsGRF6 and OsGRF8.

We found that GIFs OsGIF1, OsGIF2 and OsGIF3 interact with OsGRF4, and OsGIF3 also interacts with OsGRF6 (Fig. 5A). Knockout of *OsGIF1* led to an expected short-leaf phenotype, suggesting that miR396ef-GRFs-GIFs modules directly control the development of seeds, panicles and leaves in rice. Previous studies demonstrated that miR396 regulates seed and panicle development through brassinosteroid and auxin-signaling pathways [12,15] and our RNA-seq data revealed an altered expression of many brassinosteroid-induced genes and auxin-signaling genes in the *mir396ef* mutant (Supplementary Fig. 18). We also found that np:*rGRF4* and np:*rGRF6* have enlarged leaf angles, exhibited as losing plant structure, which are typical phenotypes of brassinosteroid pathway mutants, such as *m107* and *Gi-2* [12]. These data suggest that miR396ef might influence rice architecture by activating brassinosteroid responses.

Taken together, our findings reveal that manipulation of miR396ef can simultaneously improve grain size and panicle branching. miR396ef thus represents a promising target to increase grain size and grain yield, especially in nitrogen-deficient environments, which might help breeders develop environmentally friendly elite rice varieties.

## Materials and methods

### Plant materials and growth conditions

The rice variety *Nipponbare* (*Oryza. Sativa* L. spp. *Japonica*, var *Nipponbare*) was used in this study. Rice plants were cultivated under field conditions at two different experimental stations located in Shanghai (30°N, 121°E) and Lingshui (Hainan Province, 18°N, 110°E). The phytotron, with a 30/24 ± 1°C day/night temperature, 50%–70% relative humidity and a light/dark period of 14 hours/10 hours was used to culture rice seedlings.

### Nitrogen-deficiency response assay

Nitrogen deficiency in hydroponic culture conditions was modified from previous work [14]. Seeds were disinfected in 20% sodium hypochlorite solution for 20 min, thoroughly washed with deionized water and then germinated in a dish with sterilized water. The germinated grains were then selected and transplanted to PVC culture pots that contained hydroponic nutrient solution (0.5 mM NaH_2_PO_4_, 0.75 mM K_2_SO_4_, 1 mM CaCl_2_, 1.667 mM MgSO_4_, 40 μM Fe-EDTA (Na), 19 μM H_3_BO_3_, 9.1 μM MnSO_4_, 0.15 μM ZnSO_4_, 0.16 μM CuSO_4_ and 0.52 μM (NH_4_)_3_Mo_7_O_24_, pH 5.5) with or without 1.25 mM NH_4_NO_3_. All nutrient solutions were changed every 2 days. The temperature was maintained at 30°C day and 24°C night, and the relative humidity was 70% in phytotron.

### Vector construction

The CRISPR-Cas9 vectors targeting *MIR396abc*, *MIR396d*, *MIR396ef*, *MIR396g*, *MIR396h*, *MIR396e* and *OsGIF1* were constructed as previously described [30] and the oligos used are listed in Supplementary Table 3. To construct the miR396-resisitant vectors for *OsGRF4, OsGRF6* and *OsGRF8*, the genomic sequences of *OsGRF4*, *OsGRF6* and *OsGRF8*, fused with the *OsGRF4* promoter, *OsGRF6* promoter and *OsGRF8* promoter, respectively, were amplified and the synonymous mutations were introduced in the miR396 target sequences. All the vectors described above were used to transform rice *Nipponbare* by *Agrobacterium tumefaciens*-mediated methods.

### Phenotyping and histological experiments

Plant materials were photographed using a Canon EOS7D digital camera and observed using an OLYMPUS BX53 microscope. Grain size was analysed using an SC-A grain analysis system (Wseen Company, China).

Young rice grains were collected and fixed overnight at 4°C in FAA (50% ethanol, 10% formalin and 5% acetic acid) solution and dehydrated in a graded ethanol series. The samples were then embedded in Technovit 7100 resin (Hereaus Kulzer) and made into 2-μm sections using a Leica RM 2265 programmable rotary microtome (Leica Microsystems). After being stained with 0.05% Toluidine Blue, transverse sections were photographed using an OLYMPUS BX53 microscope. Dehydrated grain-husk samples were stocked on the copper stub and sputter-coated with gold-palladium. The seed samples were examined under a scanning electron microscope, JSM-6360LV. Cell number and cell length in the outer parenchyma layer of the spikelet hulls were measured using the OLYMPUS stream software.

### Northern blotting

Total RNA was isolated using the Trizol Reagent (Invitrogen) according to the manufacturer’s instructions. About 40 μg of total RNA from leaves and flowers were analysed on a denaturing 19% polyacrylamide gel, transferred to Nytran Super Charge Nylon Membranes (Schleicher & Schuell BioScience) and cross-linked using a Stratagene UV Crosslinker. DNA oligonucleotides complementary to different sequences of miRNAs were synthesized and labeled with [^32^P]-γ-ATP (PerkinElmer) using T4 polynucleotide kinase (TaKaRa). The membranes were pre-hybridized with PerfectHyb (Sigma) hybridization solution and then hybridized with the labeled probes. After several times of washing, the membranes were autoradiographed using an X-ray film (Carestream, X-OMAT BT Film). For RNA loading, 18S and 5S rRNAs were used as controls. The probe sequences are listed in Supplementary Table 3.

### Plot-yield tests

Plants of the WT and *mir396ef* were grown in Shanghai paddy fields under natural conditions. The area per plot was 2.0 × 2.0 m and 100 plants were cultivated in each plot with planting density of 20 × 20 cm. The nitrogen fertilizer was 270 kg ha^−1^ in the control field, whereas the nitrogen-deficient fields were without nitrogen fertilizer. Plants were irrigated with river water.

### RNA extraction and RT-qPCR and stem-loop PCR

Total RNA was isolated from roots, leaves and flowers from different developmental stages of rice plants. The RNA extraction followed the method mentioned above. After being treated with RNase-free DNase I (Promega), total RNA (1 μg) was reverse transcribed using the TransScript II One-Step gDNA Removal and cDNA Synthesis SuperMix kits (TransGen Biotech). The reverse-transcription products were used as templates for RT-qPCR performed on a CFX96 real-time PCR system (Bio-Rad) using SYBR Premix Taq (TransGen Biotech) according to the manufacturer’s protocol. *ACTIN1* was used to normalize gene expression and OsU6 was used in microRNA expression. Relative expression levels were measured using the 2^–ΔΔ*C*t^ analysis method. The primers used in RT-qPCR or stem-loop RT-qPCR are listed in Supplementary Table 3.

### Transient expression assays in *N. benthamiana* leaves

The CDS of *OSGIF1*, *OsGIF2* and *OsGIF3* were fused in frame with nYFP (yellow fluorescent protein) sequence. The CDS of *OsGRF4*, *OsGRF6* and *OsGRF8* were fused in frame with cYFP sequence. nYFP-OsGIF1, nYFP-OsGIF2, nYFP-OsGIF3, cYFP-OsGRF4, cYFP-OsGRF6 and cYFP-OsGRF8 constructs were transformed into *Agrobacteria*. These stains containing nYFP and cYFP plasmids were co-infiltrated into leaves of *N. benthamiana*. YFP signals were detected by confocal microscopy LSM800 (ZEISS). The full-length and C-terminal deletion cDNAs of *OsGRF8* were fused in frame with eGFP (enhancer Green Fluorescent Protein) sequence and driven by the 35S promoter. The stem-loop sequences of miR396e and miR396f were cloned and inserted after the 35S promoter. nYFP-OsGIF1, nYFP-OsGIF2, nYFP-OsGIF3, cYFP-OsGRF4, cYFP-OsGRF6, cYFP-OsGRF8, 35S::GRF8-eGFP, 35S::GRF8(1–574)-eGFP, 35S::GRF8(1–694)-eGFP, 35S::miR396e and 35S::miR396f constructs were transformed into *Agrobacteria*. These stains were co-infiltrated into leaves of *N. benthamiana*. YFP and GFP signals were detected by confocal microscopy LSM800 (ZEISS).

### Yeast two-hybrid assay

The prey vector pGADT7 and bait vector pGBKT7 were used in the yeast two-hybrid assay. Full-length cDNAs of *OSGIF1*, *OsGIF2*, *OsGIF3*, *OsGRF4*, *OsGRF6* and *OsGRF8* were amplified and separately cloned into pGADT7 and pGBKT7. The prey and bait plasmids were co-transformed into the yeast AH109 strain and grew on SD-Leu-Trp solid media (Clontech). The strains were transferred to the SD-Ade-His-Leu-Trp solid media to test the interactions between prey and bait.

### RLM-RACE

RLM-RACE was performed with the FirstChoice™ RLM-RACE Kit (Ambion). In general, total RNA was extracted from samples and the first and second PCRs were performed, with the primers of *OsGRF4/6/8*-inner and *OsGRF4/6/8*-outer (Supplementary Table 3), respectively. The products from the second PCR were purified by agarose gel electrophoresis and then cloned for sequencing. For *OsGRF4* and *OsGRF6*, leaf samples were used in these experiments. For *OsGRF8*, flower samples were also used in these experiments.

### Transcriptome analyses

Total RNA was extracted using the RNeasy Plant mini Kit (Qiagen) according to the manufacturer’s instructions. Two independent replicates were used for each sample. The cDNA synthesis, purification and labeling were then performed following the standard protocols. The RNA-seq data were analysed with TopHat and Cufflinks software. Genes with at least 1.5-fold up- and down-regulation in the *mir396ef* plants compared with those in WT plants were considered as DEGs.

### Net photosynthetic rate measurements

The net photosynthetic rate was measured using an LI-6400XT (LICOR Biosciences) at a light intensity of 1000 μmol m^−2^ s^−1^ and the reference CO_2_ concentration was 400 μmol mol^−1^. The measurement followed the method from the LI-6400XT manual. Two leaves were used in one measurement and placed side by side in order to fill the area of the measure room because the rice leaves were narrower than the diameter of the measure room. The net photosynthetic rate was calculated and recorded by the machine. All experiments were conducted with at least five replicates.

## Supplementary Material

nwz142_Supplemental_File
