# Supplementary material for: Disruption of MIR396e and MIR396f improves rice yield under nitrogen-deficient conditions
Source: Natl Sci Rev. 2019 Sep 27;7(1):102–12. doi: 10.1093/nsr/nwz142 (PMC8288854; doi:10.1093/nsr/nwz142)
Supplement: nwz142_Supplemental_File [file nwz142_supplemental_file.docx]

**Supplemental Figures and Figure Legends**


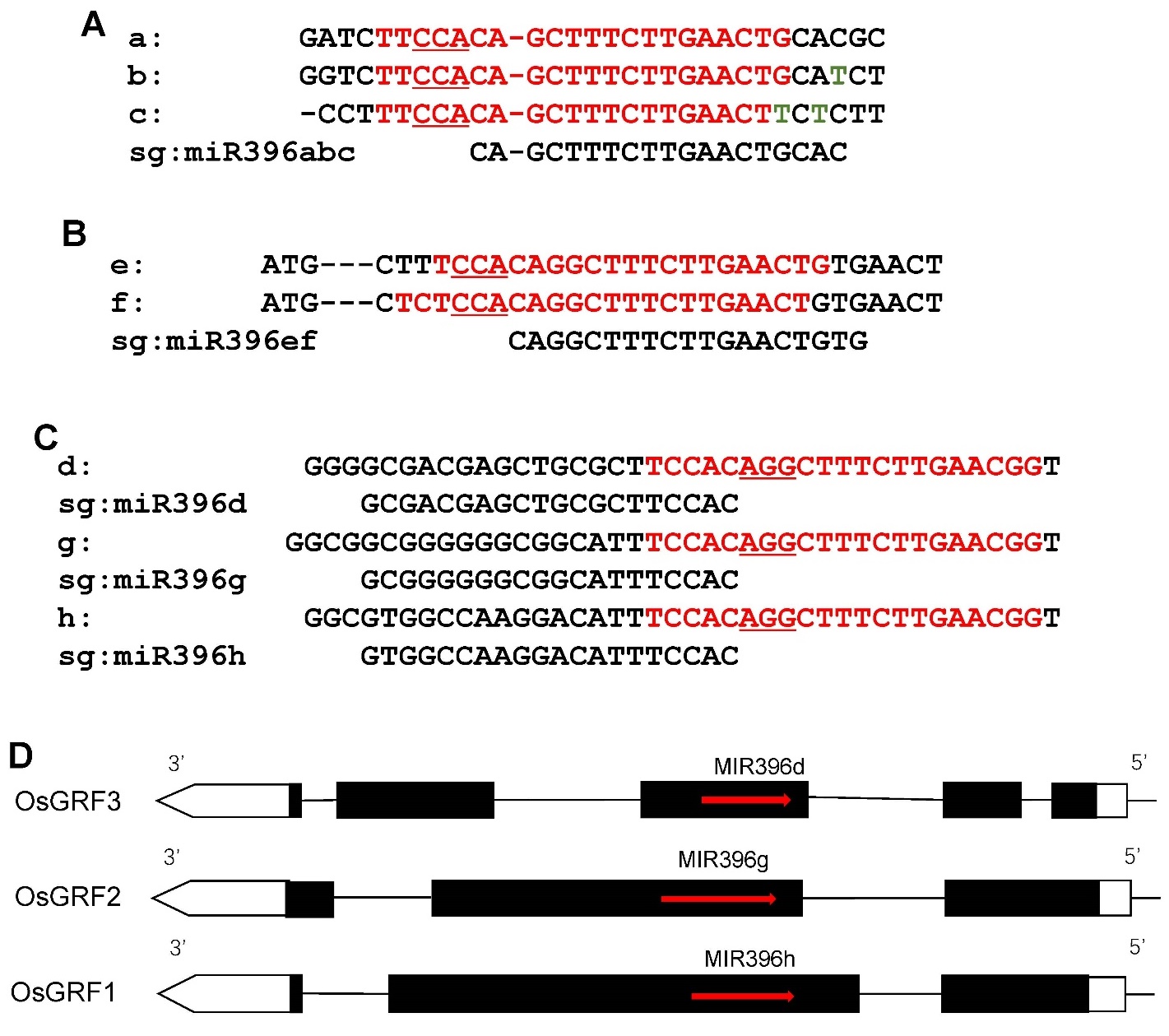


**Figure S1. CRISPR/Cas9 target sites for miR396 family members and schematic representation of *MIR396d*, *g* and *h* genes in their genomic locations.**

(A) CRISPR/Cas9 target sites for miR396a, b and c members. The green nucleotides indicate a mismatch. (B) CRISPR/Cas9 target sites for miR396e and f members. (C) CRISPR/Cas9 target sites for miR396d, g and f members. The sequences with red color correspond to mature miR396 sequence. (D) The structures of MIR396d-GRF3, MIR396g-GRF2 and MIR396h-GRF1 genes. The black boxes represent exons, untranslated regions are shown by square frame, and red arrows represent the *MIR396* genes. The PAM motif is underlined in A, B and C.


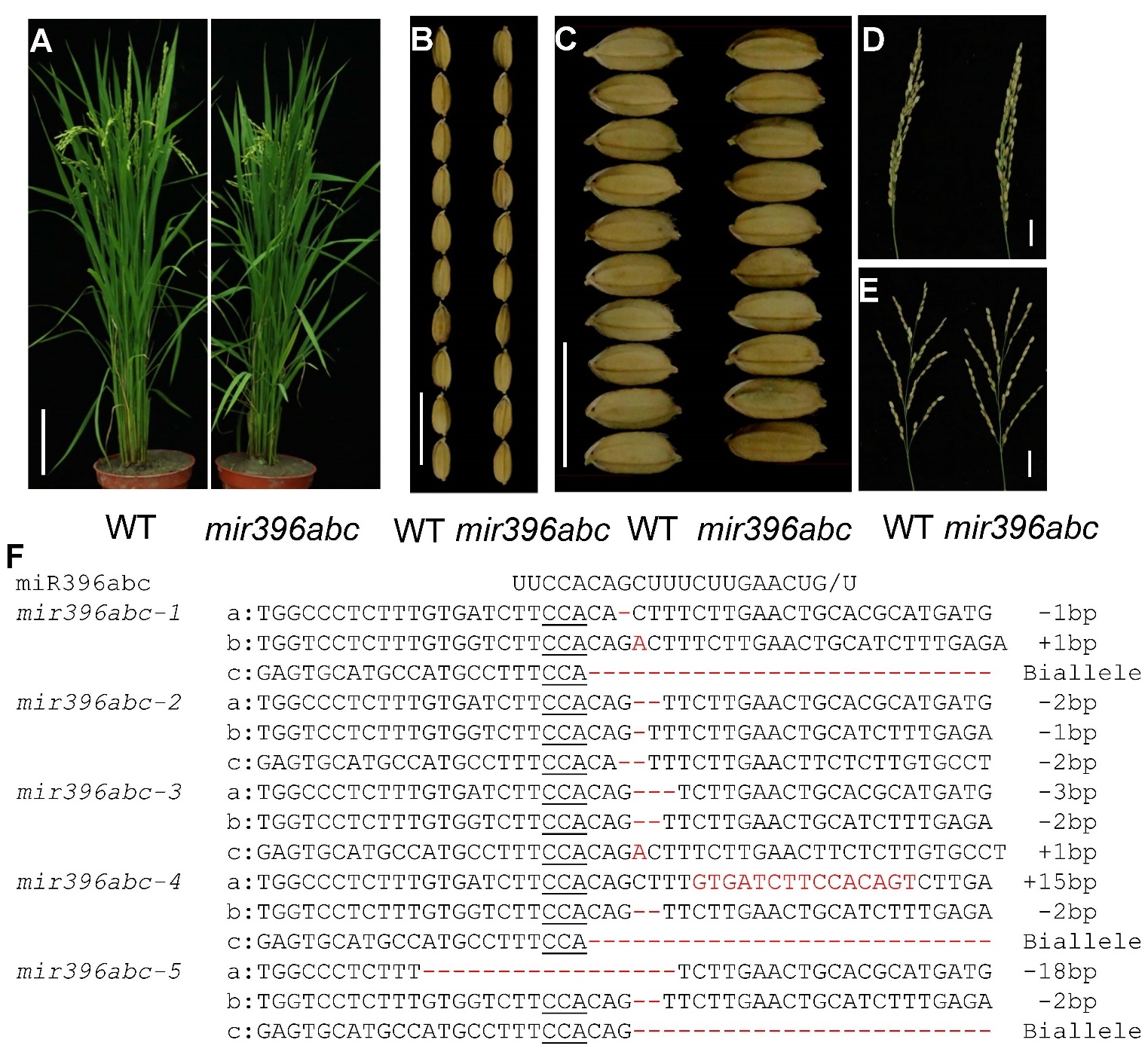


**Figure S2. Phenotypes and genotypes of *mir396abc* mutant plants.**

(A) Plant phenotypes of WT and *mir396abc* at the maturity stage. Scale bar, 10 cm. (B-C) Grain length (B) and grain width (C) of WT and *mir396abc* plants. Scale bars, 10 mm. (D-E) Panicle length (D) and panicle branching (E) of WT and *mir396abc* plants. Scale bars, 20 mm. (F) List of the genotypes of miR396a, miR396b and miR396c genes in the *mir396abc* lines. The minus (-) and plus (+) on the right indicate the numbers of nucleotides deleted and inserted at the sgRNA target site, respectively. Mutated bases are shown in red. The PAM motif is underlined.


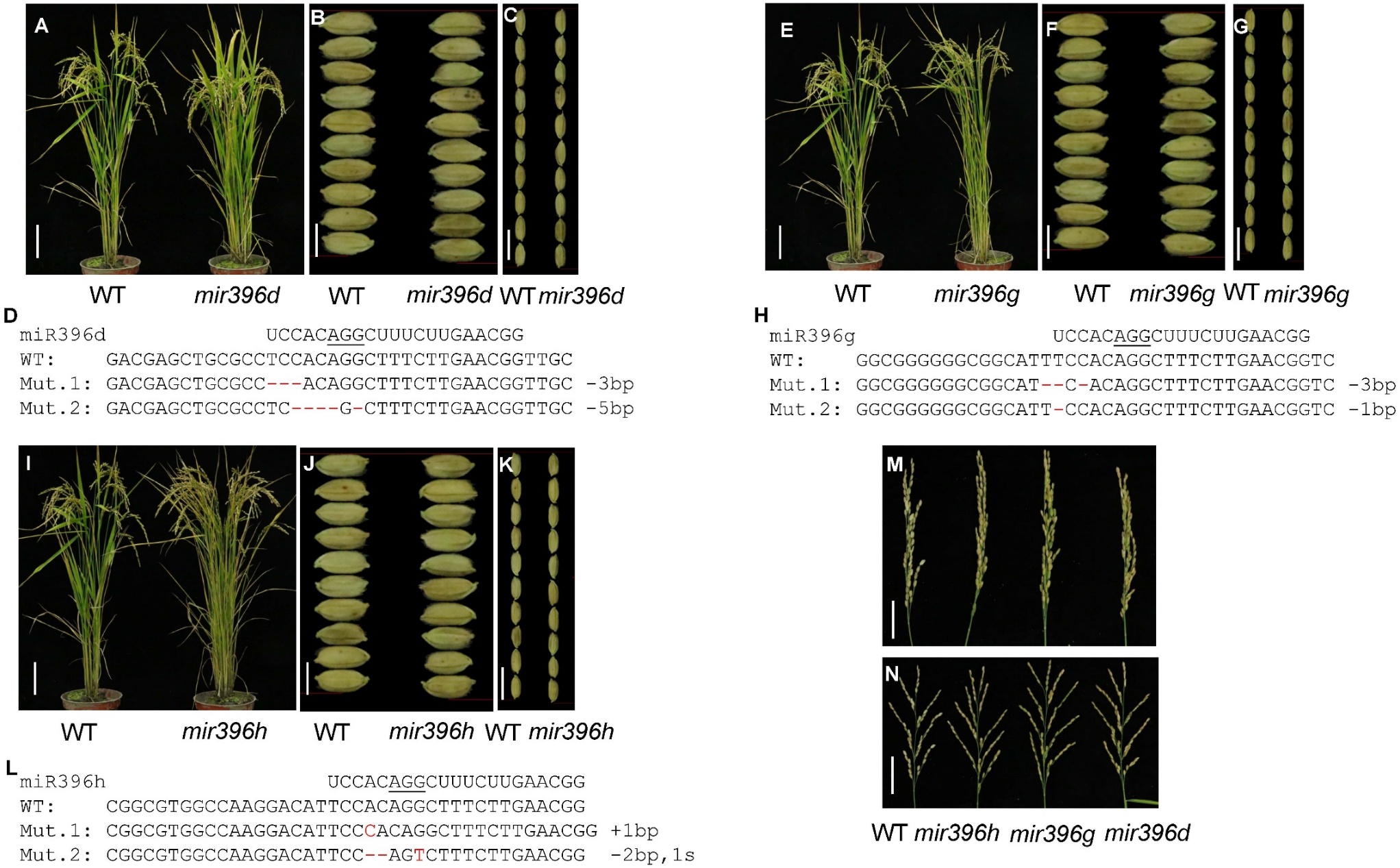


**Figures S3. Phenotypes and genotypes of *mir396d*, *g* and *h* plants.**

(A) Gross morphologies of WT and *mir396d* mutant plants. Scale bar, 10 cm. (B-C) Grain width (B) and grain length (C) of WT and *mir396d* plants. Scale bars, 5mm in B and 10mm in C. (D) Genotypes of the *mir396d* lines. (E) Gross morphologies of WT and *mir396g* mutant plants. Scale bar, 10 cm. (F-G) Grain width (F) and grain length (G) of WT and *mir396g* plants. Scale bars, 5mm in F and 10mm in G. (H) Genotypes of the *mir396g* lines. (I) Gross morphologies of WT and *mir396h* mutant plants. Scale bar, 10 cm. (J-K) Grain width (J) and grain length (K) of WT and *mir396h* plants. Scale bars, 5mm in J and 10mm in K. (L) Genotypes of the *mir396h* lines. (M-N) The panicle length (M) and panicle branching (N) of WT and *mir396d, g, h* plants. Scale bars, 5 cm. The minus (-) and plus (+) indicate the numbers of nucleotides deleted and inserted at the sgRNA target site, respectively. The “1s” indicates one nucleotide substitution. Mutated bases are shown in red. The PAM motif is underlined.


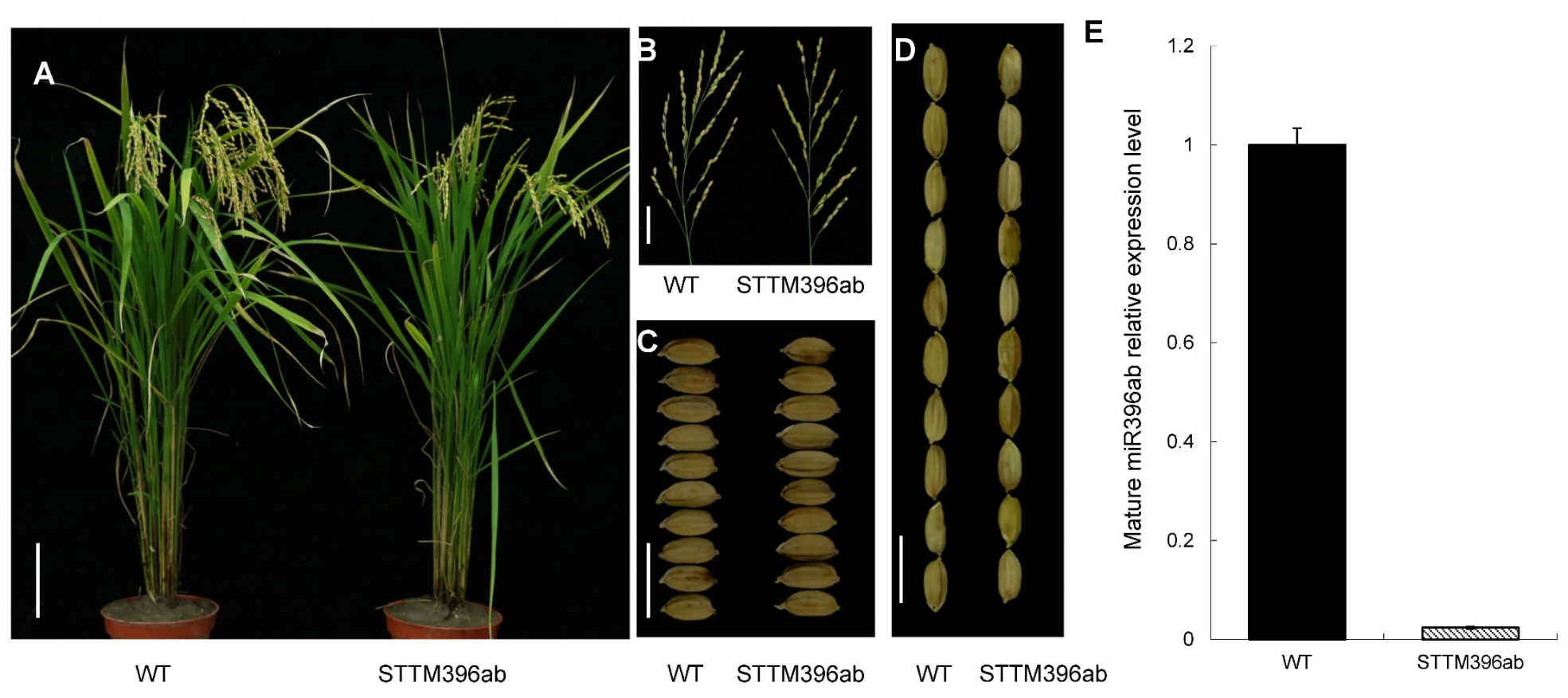


**Figure S4. The phenotypes of STTM396ab plants and the expression of miR396ab in WT and STTM396ab plants.**

(A) Morphologies of WT and STTM396ab plants. Scale bar, 10 cm. (B) Panicle branching of WT and STTM396ab plants. Scale bar, 5 cm. (C-D) Grain width (C) and grain length (D) of WT and STTM396ab. Scale bars, 10 mm. (E) Expression level of miR396ab in WT and STTM396ab plants assayed by stem-loop RT-qPCR. U6 snRNA was used to normalize the samples. Data are presented as means ±SD, n=3.


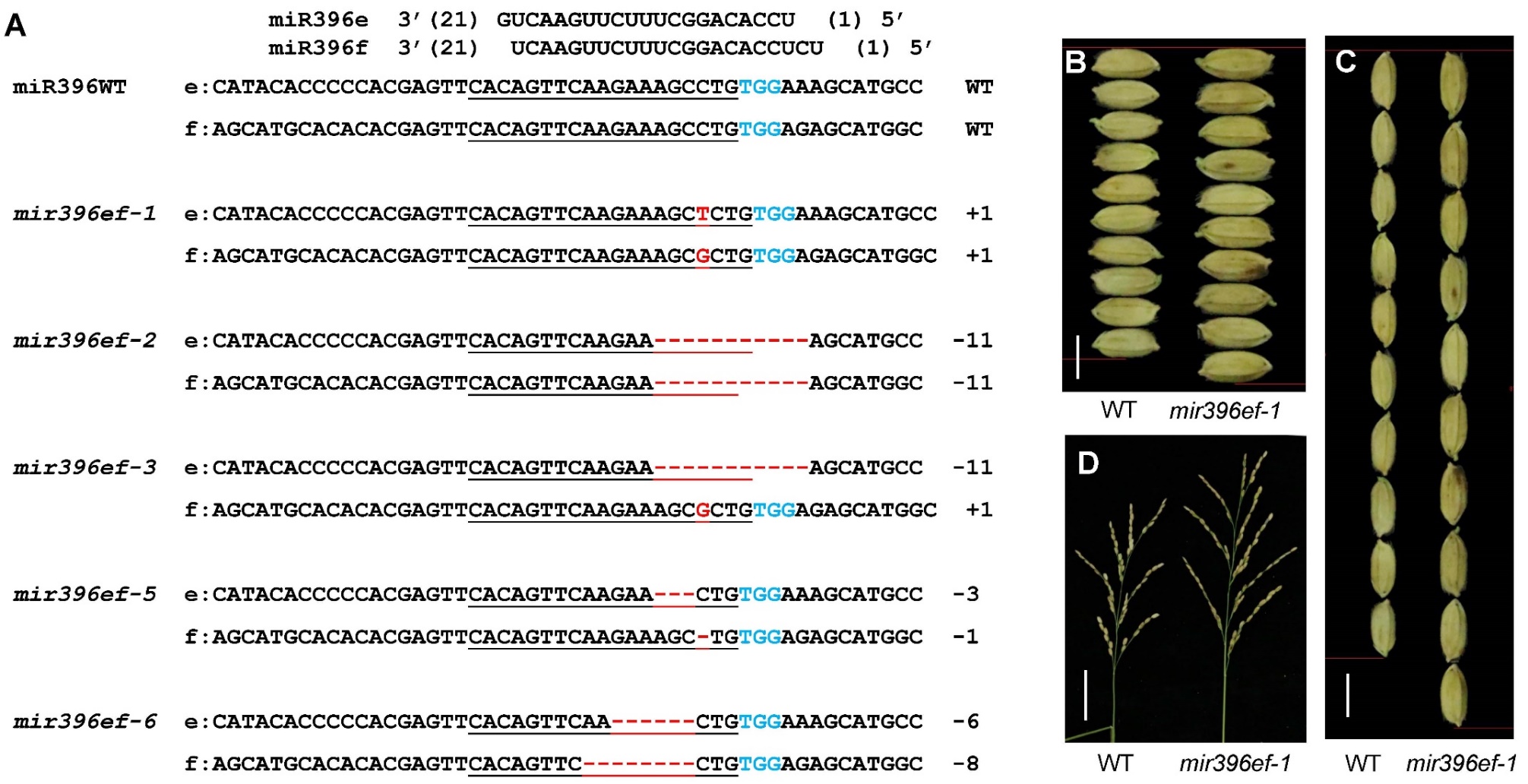


**Figure S5. Genotypes and phenotypes of *mir396ef* plants.**

(A) List of the genotypes of miR396e and miR396f in *mir396ef* plants. On the right, minus (-) and plus (+) indicate the number of nucleotides deleted and inserted at the sgRNA target site, respectively. Mutated bases are shown in red. The PAM motif is in blue. (B-D) Grain width (B), grain length (C) and panicle branching (D) of WT and *mir396ef-1* plants. Scale bars, 5 mm in B and C, and 5cm in D.


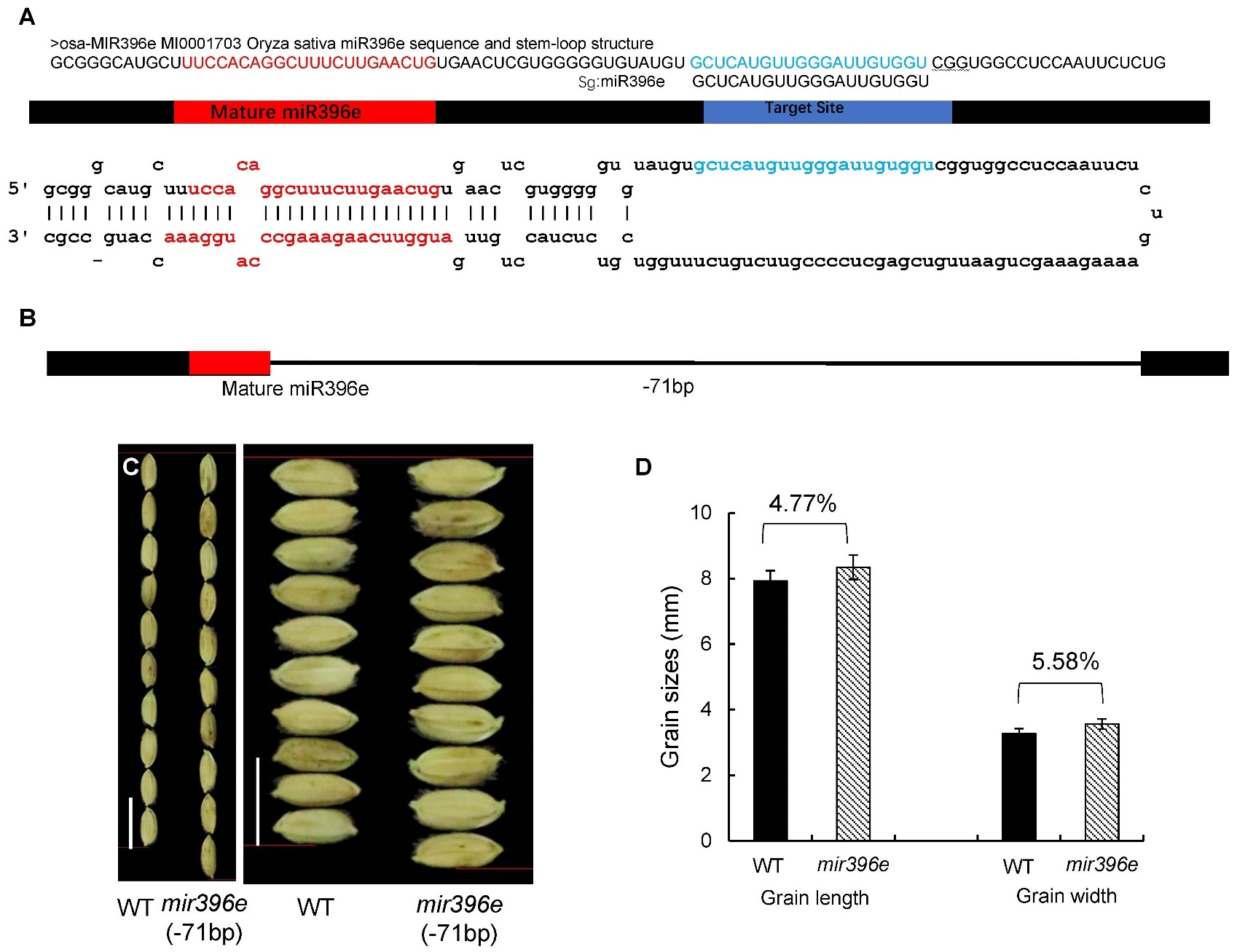


**Figure S6. Schematic representation CRISPR/Cas9 target sites for miR396e and phenotypes of *mir396e*.**

(A) The precursor sequence and schematic secondary structure of miR396e. CRISPR/Cas9 target site is highlighted in blue. Mature sequence of miR396e is highlighted in red. (B) The deletion region in the precursor sequence of *mir396e* mutant. (C) Grain length (left) and grain width (right) of WT and *mir396e*. Scale bars, 10 mm. (D) Statistical data for grain sizes of WT and *mir396e*. Data are presented as means ± SD (n=100 in D).


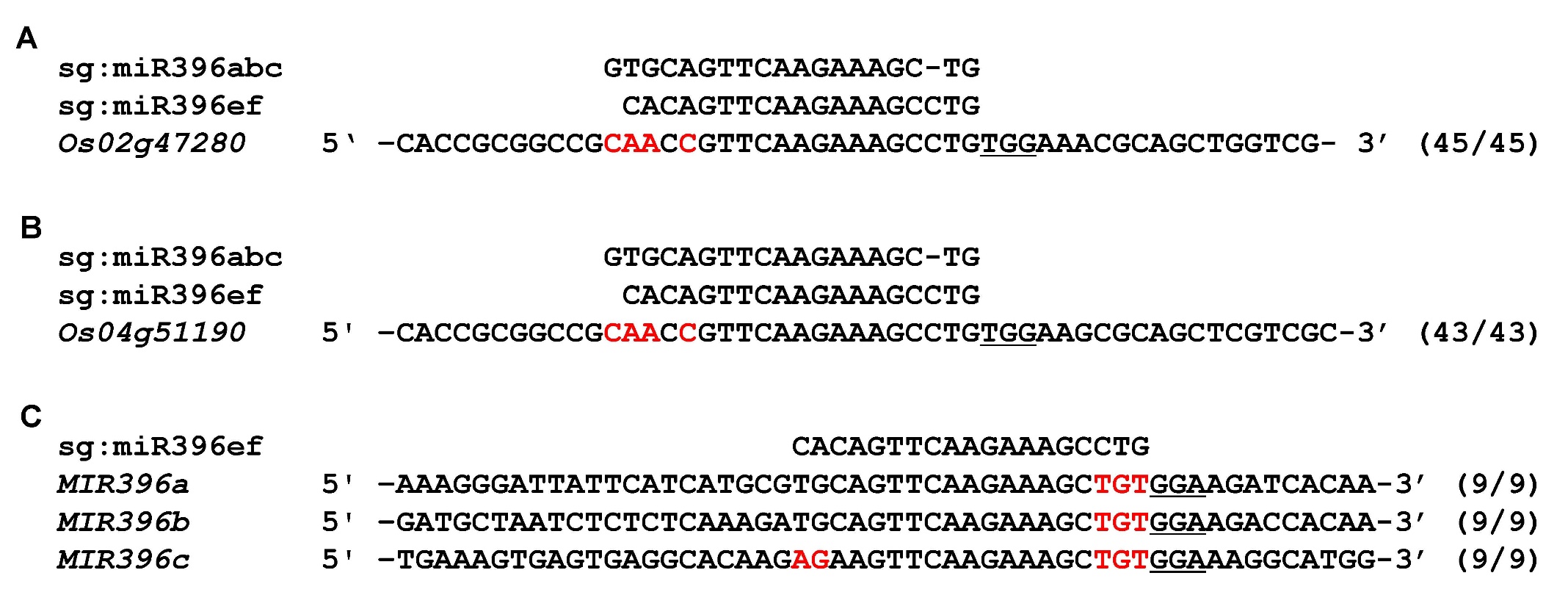


**Figure S7. Analysis of putative off-target sites.**

(A) Predicted sg:miR396abc and sg:miR396ef off-target sites in *LOC_Os02g47280* gene. (B) Predicted sg:miR396abc and sg:miR396ef off-target sites in *LOC_Os04g51190* gene. (C) Predicted sg:miR396ef off-target sites in *MIR396a*, *MIR396b* and *MIR396c* genes. The mismatched bases are shown in red. The PAM motif is underlined. The numbers in bracket indicates the numbers of WT lines/total tested lines.


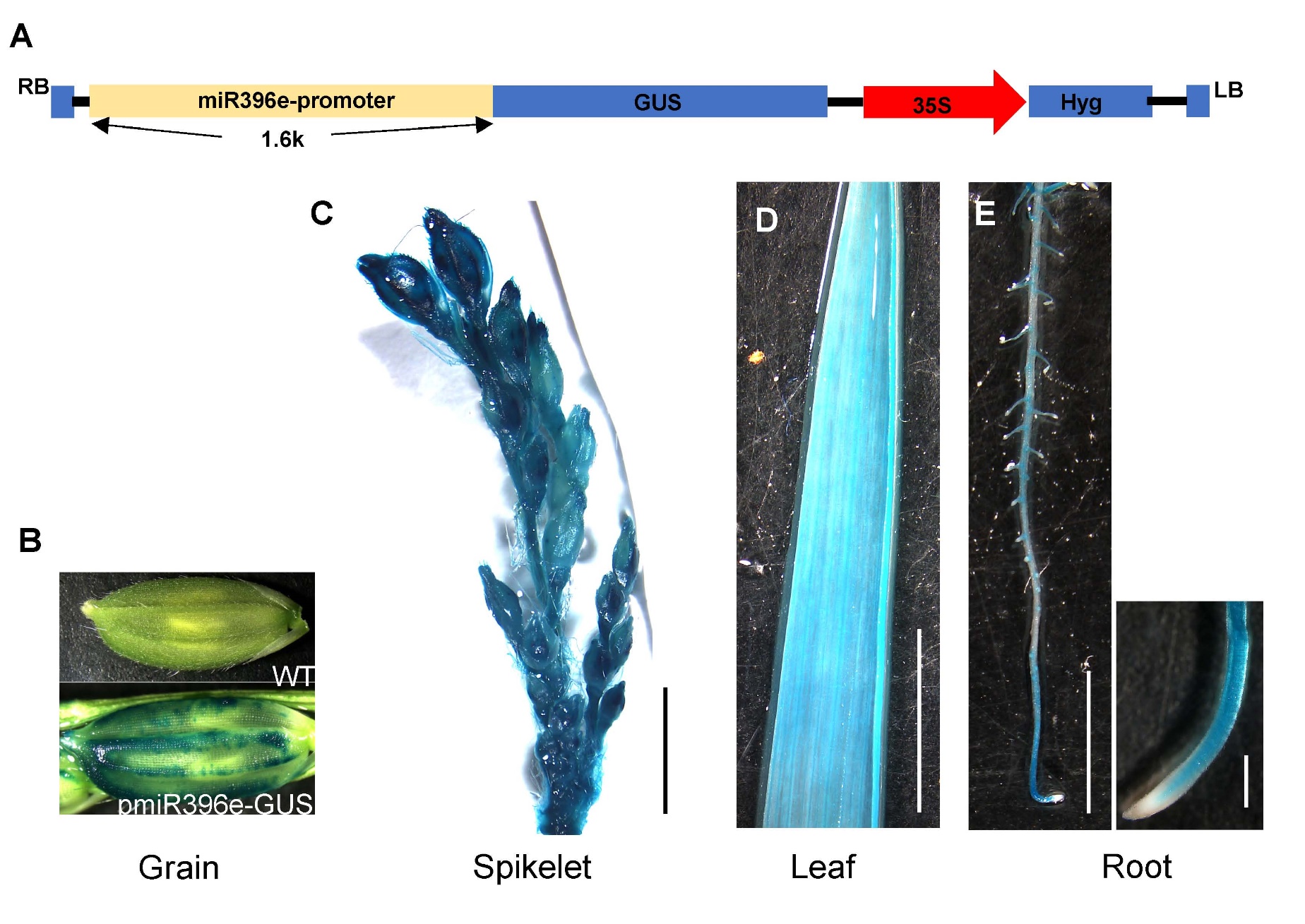


**Figure S8. miR396e promoter-driven GUS expression in grain, spikelet, leaf and root of rice.**

(A) Schematic diagram of the miR396e promoter:GUS vector. (B-E) GUS staining in grain husk (B), spikelet (C), leaf (D) and root (E). Scale bars, 2.0 cm in C, D and E (Left) and 50 mm in E (Right).


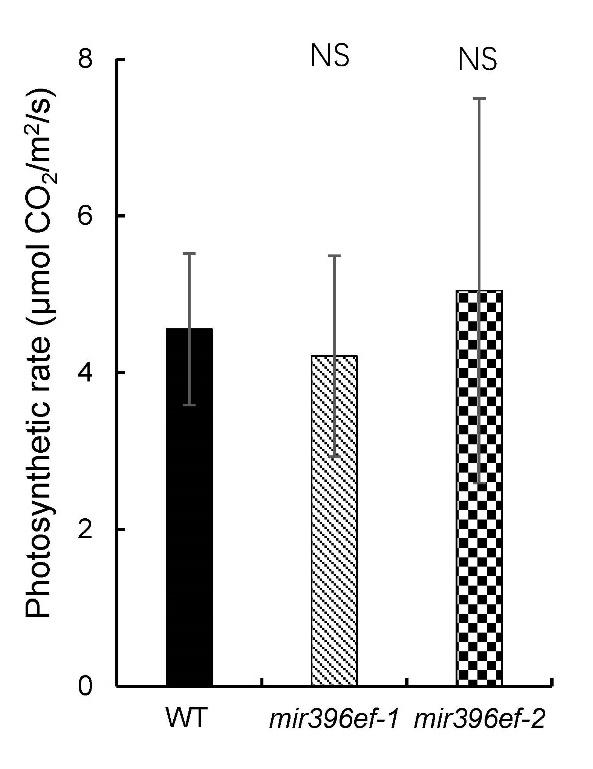


**Figure S9. Photosynthetic rate of WT and *mir396ef* plants grown in the paddy field.**

“(μmol CO_2_/m^2^/s)” represents passing CO_2_ in unit area per second through the leaf blade. Data are presented as means ±SD. n=5. NS: Not Significant, two-tailed, two sample t test.


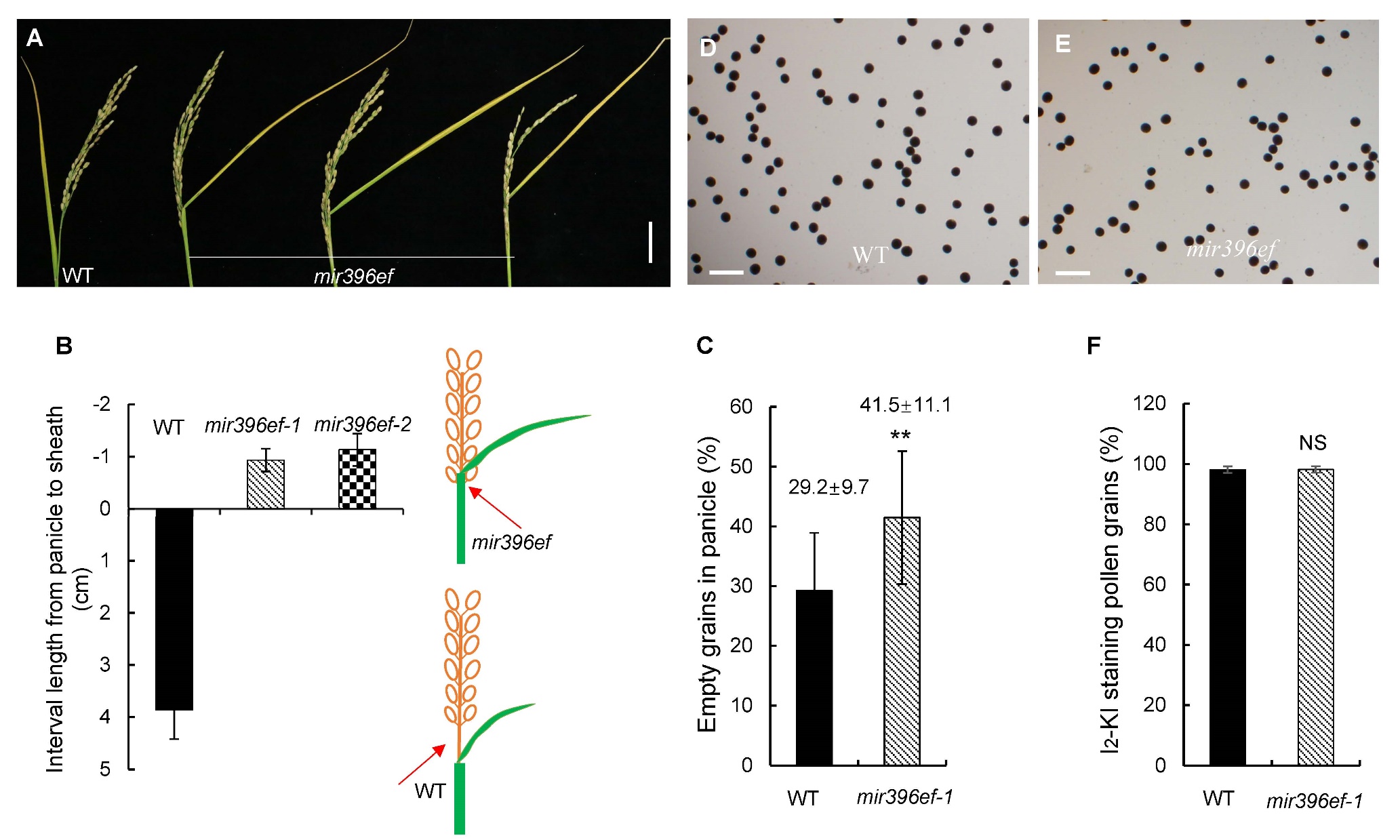


**Figure S10. Phenotypes of flag leaf sheaths at maturity and empty grains in panicles of *mir396ef* plants.**

(A) Phenotypes of flag leaf sheaths with panicles of WT and *mir396ef* plants. Scale bar, 5 cm. (B) Interval length from panicle to sheath of WT and *mir396ef* at maturity. (C) The rate of empty grains in panicle of WT and *mir396ef* plants. (D-E) I_2_-KI staining of pollen grains from WT (D) and *mir396ef* (E). Bars, 100 μm in D and E. (F) The percentage of I_2_-KI stained pollen grains of WT and *mir396ef* plants. Data are given as means ±SD. Student’s t-test was used to generate the P values; **P < 0.01, NS, not significant.


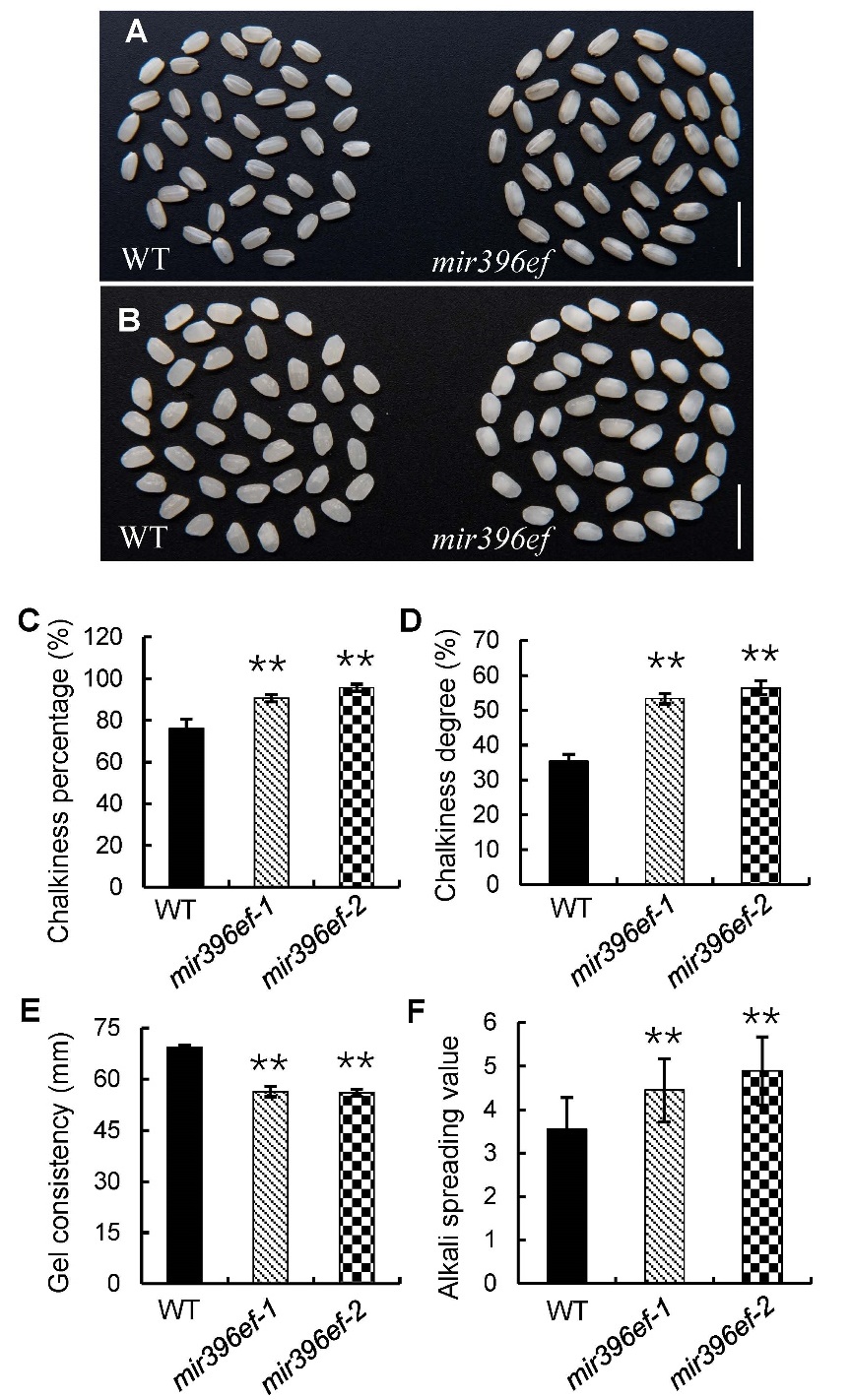


**Figure S11. Phenotypes of *mir396ef* grain quality traits.**

(A-B) Brown (A) and polished (B) grains of WT and *mir396ef* plants. Scale bars, 10 mm in A and B. (C–F) Statistical data for chalkiness percentage (C), chalkiness degree (D), gel consistency (E), and alkali spreading value (F). Data are given as means ±SD (n=9 in C-F). Student’s t-test was used to generate the P values; **P < 0.01.


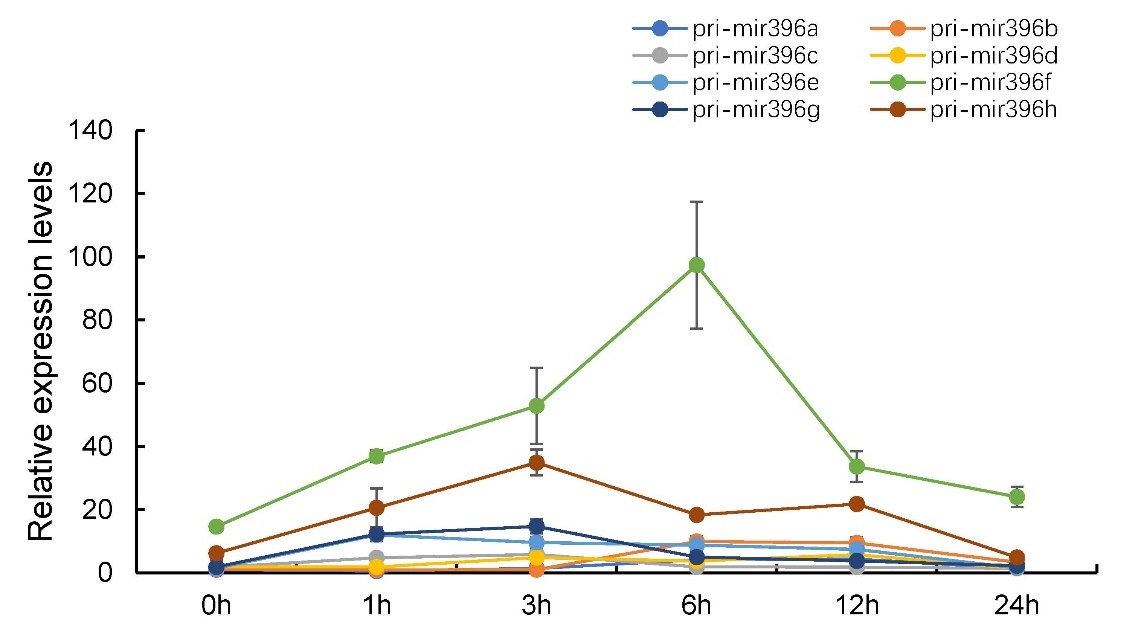


**Figure S12. Relative expression levels of miR396 family members under normal and nitrogen-deficient conditions.**

The transcript levels of all eight pri-miR396 members in WT seedling samples were detected by RT-qPCR. ACTIN1 was used for normalization; relative expression levels were measured using the 2^-△△Ct^ analysis method.


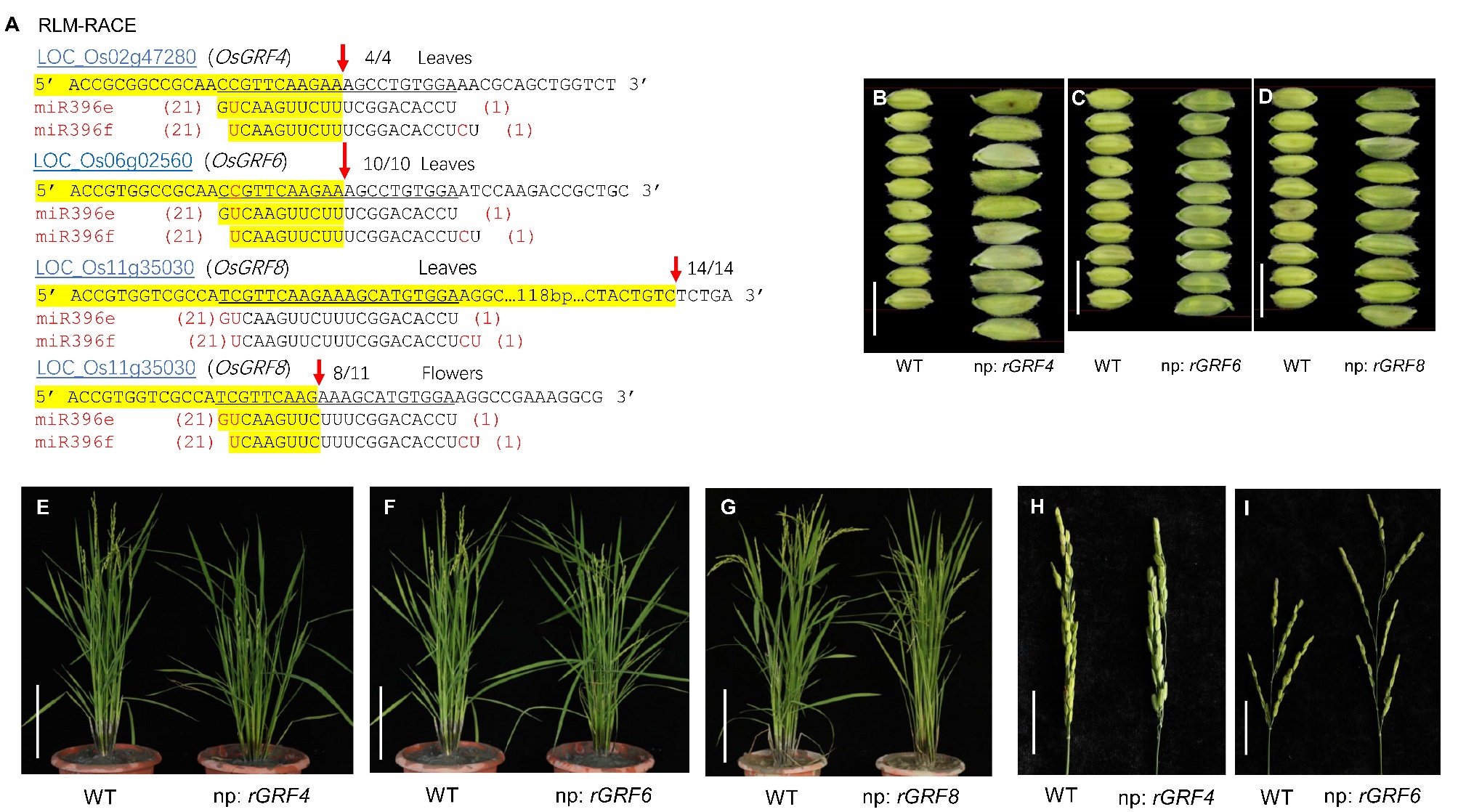


**Figure S13. Phenotypes of *rOsGRF4*, *rOsGRF6* and *rOsGRF8* plants.**

(A) miR396ef cleavage sites in *OsGRF4*, *OsGRF6* and *OsGRF8* mRNAs. The positions corresponding to the 5’ ends of the cleaved *OsGRF4*, *OsGRF6* and *OsGRF8* mRNAs determined by 5’ RACE and the numbers of 5’ RACE clones corresponding to each site are shown beside the arrows. The mismatched bases are shown in red. Red arrow indicates the cleavage site detected by 5’-RACE experiments. Leaves and flowers indicated the materials used in different experiments. (B-D) Grain width of WT and np: *rOsGRF4* (B), np: *rOsGRF6* (C) and np:*rOsGRF8* (D) respectively. Scale bars, 10 mm. (E-G) Gross morphologies of WT and np: *rOsGRF4* (E), np: *rOsGRF6* (F) and np: *rOsGRF8* (G) plants at maturity respectively. Scale bars, 20 cm. (H) Phenotypes of panicle in WT and np: *rOsGRF4* plants. Scale bar, 5 cm. (I) Phenotypes of panicle branching of WT and np: *rOsGRF6*. Scale bar, 5 cm.


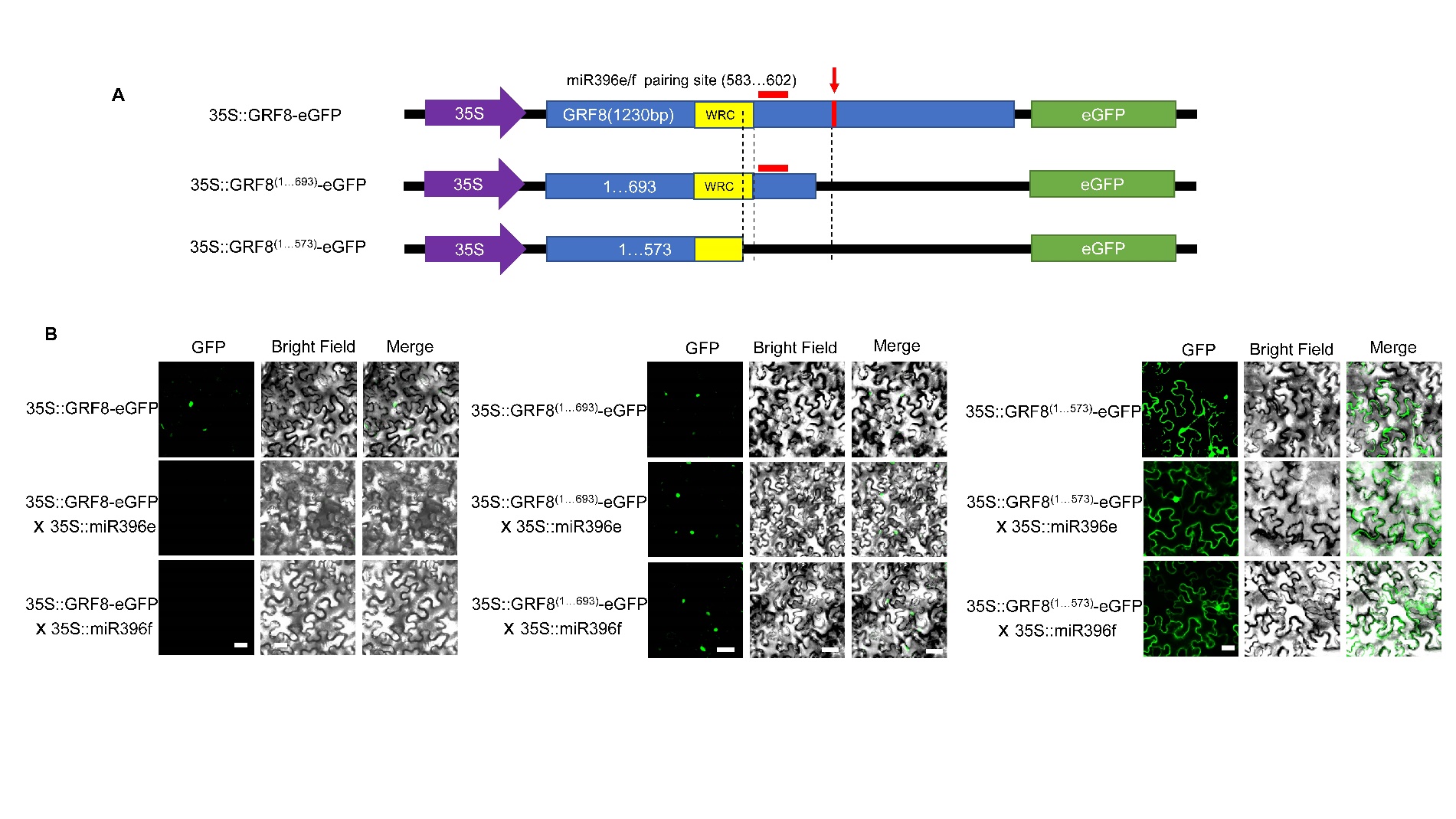


**Figure S14. Effects of rice miR396e and miR396f on *OsGRF8* in transient expression assays in *N. benthaminana* leaves.**

1. Scheme of the full-length and C-terminal deletion cDNAs of *OsGRF8* fused in frame with eGFP (enhancer Green Fluorescent Protein) sequence and driven by the 35S promoter. GRF8(1…1230), the full-length cDNA sequence of *OsGRF8*; GRF8^(1…693)^, with deletion of the 3’ region including the miR396 cleavage site detected by 5’-RACE experiments with leaf samples; GRF8^(1…573)^, with deletion of the 3’ region including the miR396 cleavage site detected by 5’-RACE experiments with leaf samples and the predicted miR396 pairing site. The stem-loop sequences of miR396e and miR396f were cloned and inserted after 35S promoter in another vector. The WRC domain contains a putative nuclear localization signal and a DNA-binding domain. In GRF8^(1…573)^, the WRC domain is destroyed, which affects the nuclear localization of OsGRF8. The red arrow indicates the cleavage site detected by 5’-RACE experiments with leaf samples. **(B)** Analysis of miR396e and miR396f effects on OsGRF8-GFP, OsGRF8^(1…573)^ and OsGRF8^(1…693)^ fluorescence in transiently transformed *N. benthamiana* leaf epidermal cells. Scale bars, 40μm.


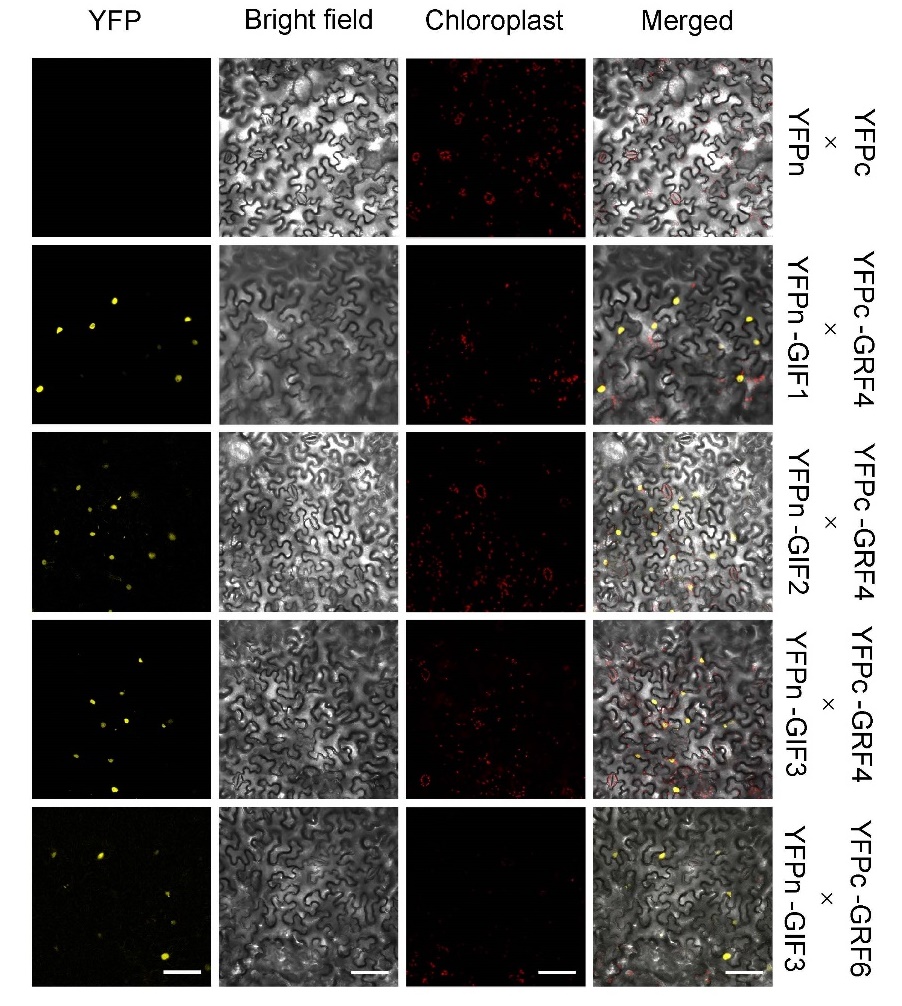


**Figure S15. BiFC assays showing the interactions between OsGIFs and OsGRFs.**

Scale bars, 50 μm.


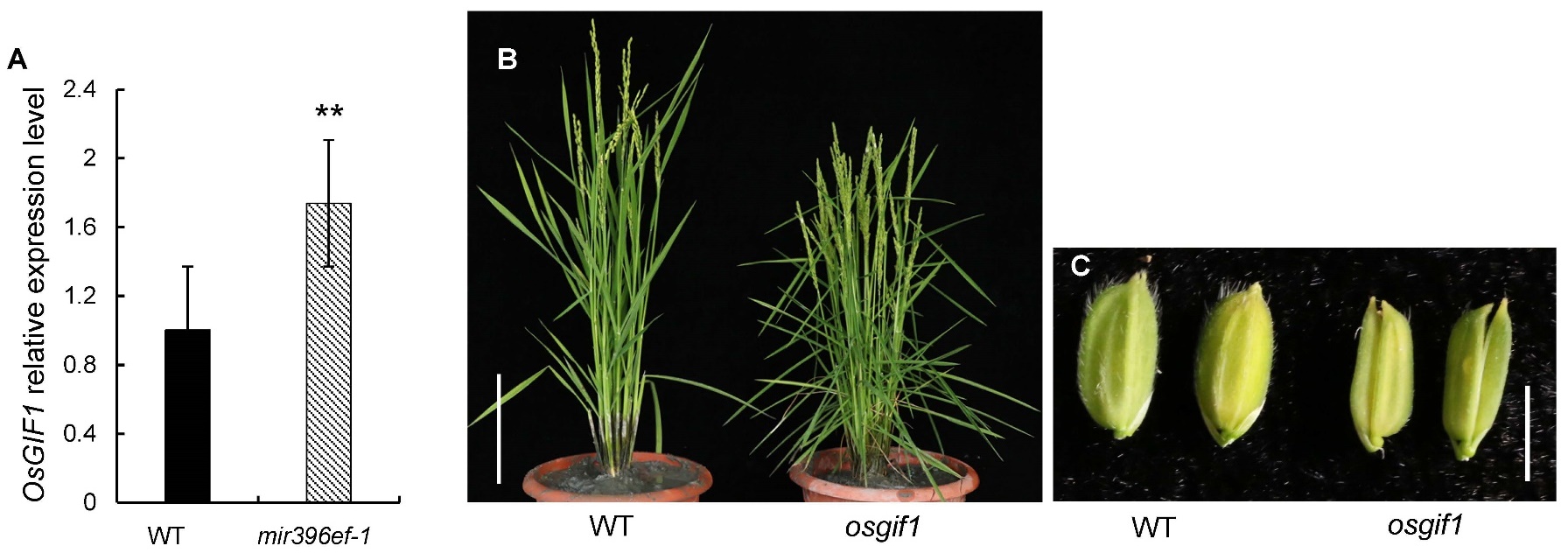


**Figure S16. Expression levels of *OsGIF1* in WT and *mir396ef* mutant and phenotypes of *osgif1* plants.**

(A) Expression level of *OsGIF1* in WT and *mir396ef* mutant determined by RT-qPCR. ACTIN1 was used for normalization; relative expression level was measured using the 2^-△△Ct^ analysis method. Data are given as means ± SD (n=3). Student’s t-test was used to generate the P values; **P < 0.01. (B) Morphologies of WT and *osgif1* at the mature stage. Scale bar, 20 cm. (C) Grain phenotypes of WT and *osgif1*. Scale bar, 5 mm.


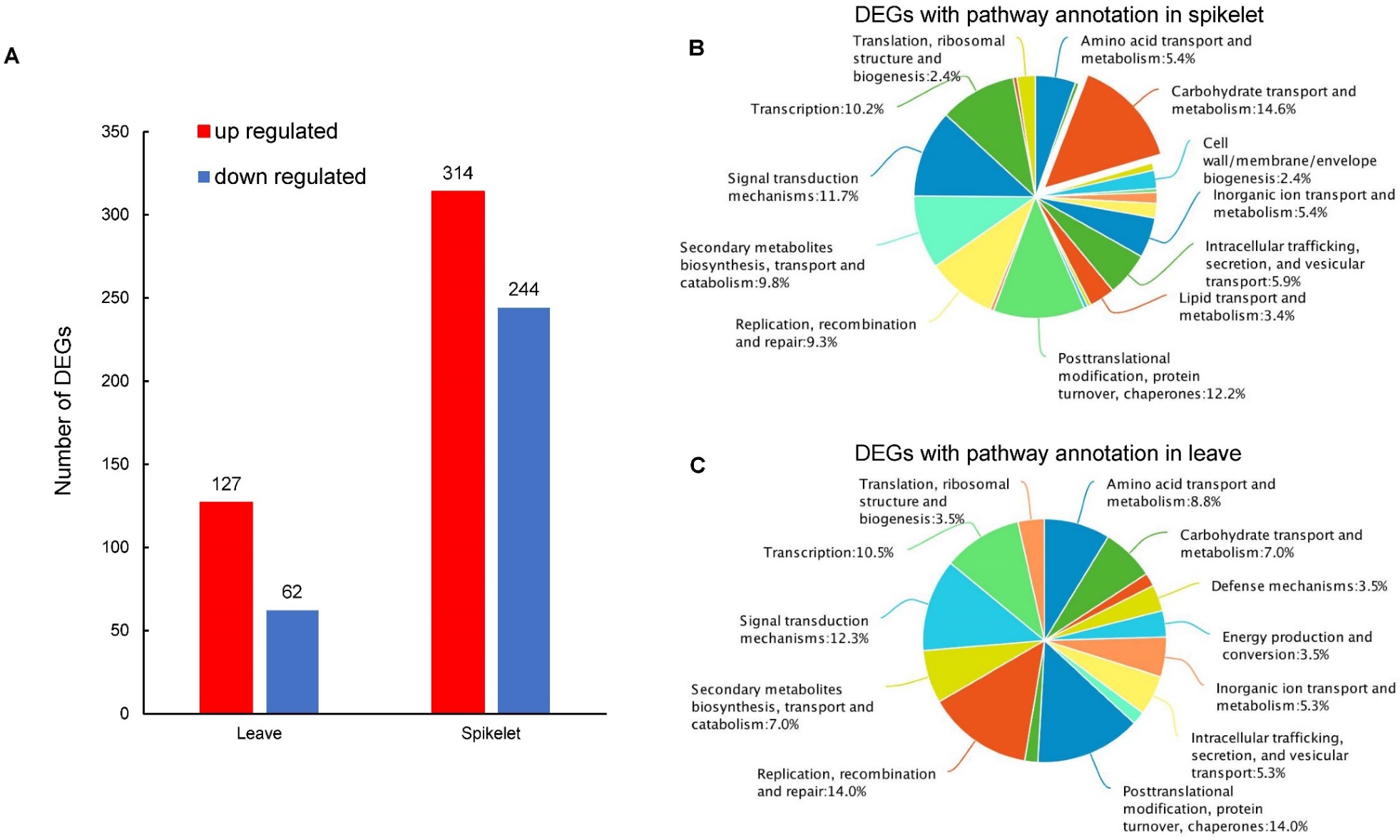


**Figure S17. Distribution of DEGs in *mir396ef* vs WT.**

(A) DEG (>1.5 fold) numbers of *mir396ef* vs WT of spikelet and leaf tissues. (B) Enriched DEG numbers with annotation in known pathways in spikelet. (C) Enriched DEG numbers with annotation in known pathways in leaves.


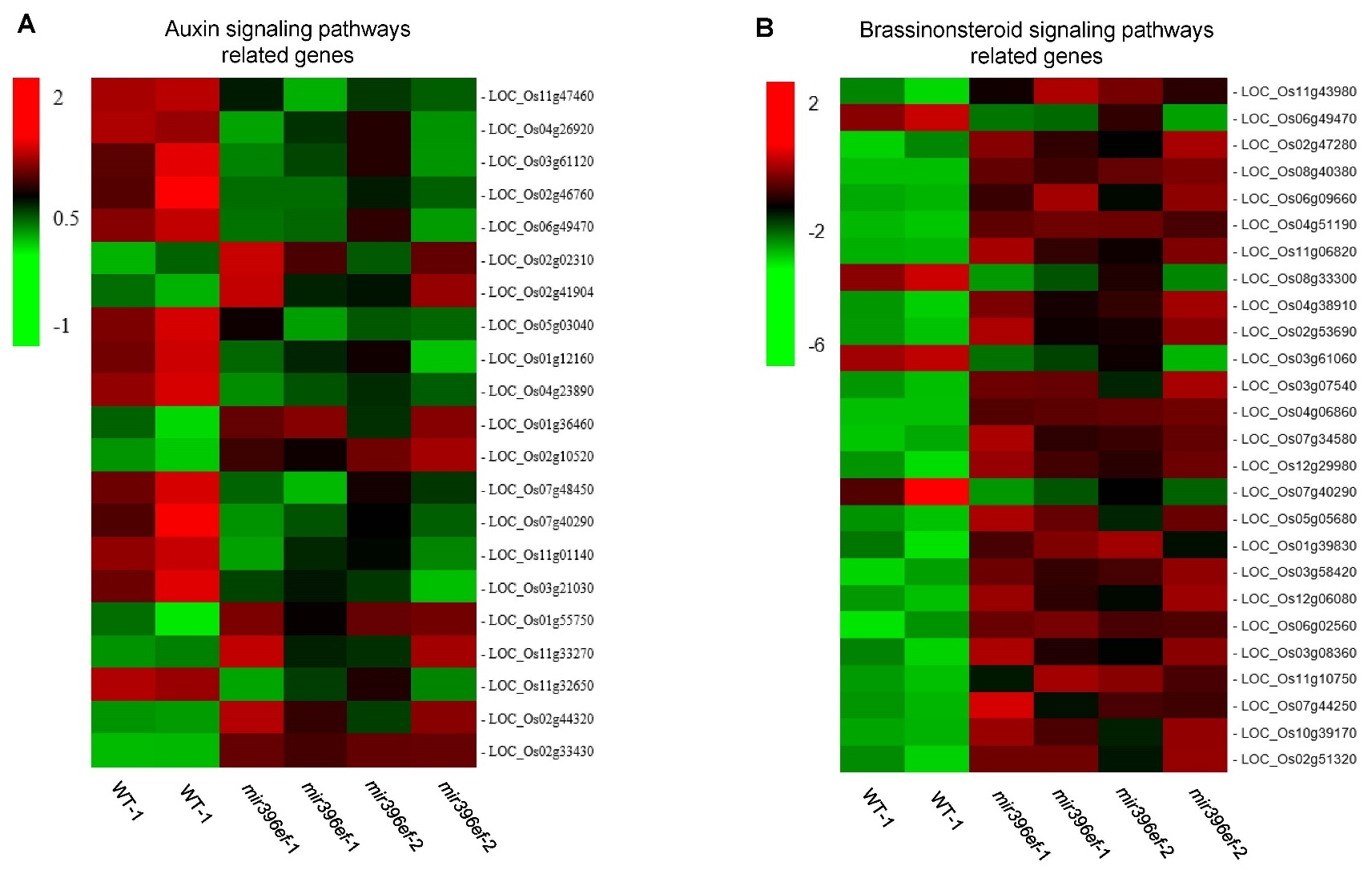


**Figure S18. Heatmap of genes related to brassinosteroid and auxin signaling** pathways **in spikelets.**

(A) The heatmap of genes related to auxin signaling pathways. (B) The heatmap of genes related to brassinosteroid signaling pathways.


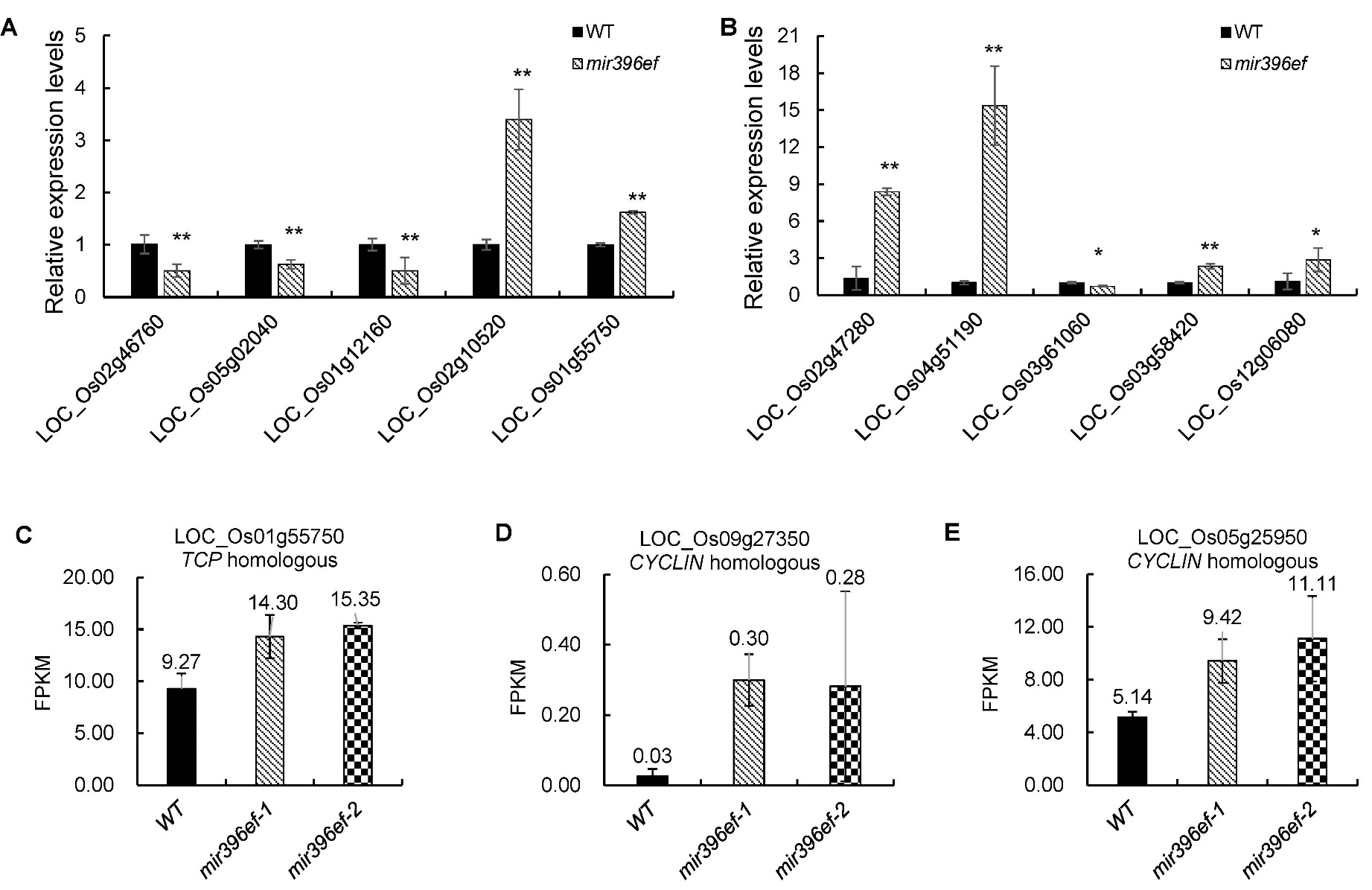


**Figure S19. Relative expression levels of genes in WT and *mir396ef* mutants determined by RT-qPCR and RNA-seq data.**

(A-B) Relative expression levels of genes related to auxin (A) and brassinosteroid (B) signaling pathways in WT and *mir396ef* samples detected by RT-qPCR. ACTIN1 was used for normalization; relative expression levels were measured using the 2^-△△Ct^ analysis method. Data are given as means ± SD (n=3). Student’s t-test was used to generate the P values; *P < 0.05, **P < 0.01. (C)The Fragments Per Kilobase of transcript per Million mapped reads (FPKM) of *TCP4* homolog gene *LOC_Os01g55750* in spikelet tissue of WT and *mir396ef* determined by RNA-seq. (D-E) The FPKM of *CYCINB1;1* homologous gene *LOC_Os09g27350* (D) and *LOC_Os05g25905* (E) in spikelet tissue of WT and *mir396ef* determined by RNA-seq.

**Table S1. Expression of miR396 members in reproductive tissues.**

|  | **RPM** (the reads per million miRNA reads) | | |
| --- | --- | --- | --- |
|  | **Inflorescence** | **Florets** | **Spikelets** |
| *[Osa-MIR396a](http://www.pmiren.com/singlemirna?Accession=PmiREN014328)* | 242.3899359 | 33.20612077 | 3.725233181 |
| *[Osa-MIR396b](http://www.pmiren.com/singlemirna?Accession=PmiREN014329)* | 242.3899359 | 33.20612077 | 3.725233181 |
|  |  |  |  |
| *[Osa-MIR396c](http://www.pmiren.com/singlemirna?Accession=PmiREN014330)* | 1035.680068 | 178.586828 | 4.268660749 |
|  |  |  |  |
| *[Osa-MIR396d](http://www.pmiren.com/singlemirna?Accession=PmiREN014331)* | 9.874755483 | 0.301879554 | 0.027624574 |
| ***[Osa-MIR396e](http://www.pmiren.com/singlemirna?Accession=PmiREN014332)*** | **11858.61487** | **700.7333095** | **33.90010159** |
|  |  |  |  |
| ***[Osa-MIR396f](http://www.pmiren.com/singlemirna?Accession=PmiREN014333)*** | **11863.44246** | **701.1283382** | **33.90748086** |
|  |  |  |  |
| *[Osa-MIR396g](http://www.pmiren.com/singlemirna?Accession=PmiREN014334)* | 9.874755483 | 0.301879554 | 0.027624574 |
|  |  |  |  |

**Data from the online plant miRNA database PmiREN (Plant miRNA**

**ENcyclopedia) (http://www.pmiren.com/).**

**Table S2. Grain yields of WT and *mir396ef* in the control and low nitrogen**

**paddy fields.**

|  | **Cultivation conditions** | **Grain yield (kg per plot)** | **Yield decrease in low nitrogen** |
| --- | --- | --- | --- |
| **WT** | Control | 2.73 ± 0.15 | 16.29% |
|  | Low nitrogen | 2.29 ± 0.17 |  |
| ***mir396ef*** | Control | 2.84 ± 0.16 | 7.47% |
|  | Low nitrogen | 2.63 ± 0.17 |  |

**Table S3. List of primers used in this study.**

| Description | Primers name | Sequences (5'-3') |
| --- | --- | --- |
| CRISPR/Cas9 target to MIR396s | miR396abc-sg-F | TGTGTGGTGCAGTTCAAGAAAGCTG |
|  | miR396abc-sg-R | AAACCAGCTTTCTTGAACTGCACCA |
|  | miR396def-sg-F | TGTGTGCACAGTTCAAGAAAGCCTG |
|  | miR396def-sg-R | AAACCAGGCTTTCTTGAACTGTGCA |
|  | miR396d-sg-F | TGTGTGCGGGGGGCGGCATTTCCAC |
|  | miR396d-sg-R | AAACGTGGAAATGCCGCCCCCCGCA |
|  | miR396g-sg-F | TGTGTGCGACGAGCUGCGCTTCCAC |
|  | miR396g-sg-R | AAACGTGGAAGCGCAGCTCGTCGCA |
|  | miR396h-sg-F | TGTGTGTGGCCAAGGACATTTCCAC |
|  | miR396h-sg-R | AAACGTGGAAATGTCCTTGGCCACA |
|  | miR396e-sg-F | TGTGTGCTCATGTTGGGATTGTGGT |
|  | miR396e-sg-R | AAACACCACAATCCCAACATGAGCA |
| CRISPR/Cas9 target to GIF1 | GIF1-Crispr-F | TGTGTGACAACCAGAACAATGGGA |
|  | GIF1-Crispr-R | AAACTCCCATTGTTCTGGTTGTCA |
| RT-qPCR | stem-loop-miR396a | GTTGGCTCTGGTGCAGGGTCCGAGGTATTCGCACCAGAGCCAACCAGTTC |
|  | Forward-miR396a | GCGGCGGTTCCACAGCTTTCTT |
|  | stem-loop-miR396e | GTTGGCTCTGGTGCAGGGTCCGAGGTATTCGCACCAGAGCCAACCAGTTC |
|  | Forward-miR396e | GCGGCGGTCCACAGGCTTTCTT |
|  | stem-loop-miR396f | GTTGGCTCTGGTGCAGGGTCCGAGGTATTCGCACCAGAGCCAACAGTTCT |
|  | Forward-miR396f | GCGGCGGTCTCCACAGGCTTTCT |
|  | universal primer | GTGCAGGGTCCGAGGT |
| Northern blotting | miR396f-probe | AGTTCAAGAAAGCCTGTGGAGA |
|  | miR396e-probe | CAGTTCAAGAAAGCCTGTGGA |
| Detecting MIR396 mutations | miR396a-DF | TCCTTTCGATAGCGGTGCAG |
|  | miR396a-DR | GGCGCATTTGAATGTTAGGG |
|  | miR396b-DF | CATGAGCACCTGAACTATACAA |
|  | miR396b-DR | AACAATGGACCCAACAAGAC |
|  | miR396c-DF | CGTAAGCAAAAAGGGCACCAA |
|  | miR396c-DR | ATGGCATGGAGAGGTGTTGC |
|  | miR396e-DF | CCACCTCTGCATCTTCTACTT |
|  | miR396e-DR | CCAAATTCAGAACCAAAACC |
|  | miR396f-DF | TTACCTGCTAGATCGAATTG |
|  | miR396f-DR | ATACCACCCCTCTTTCTCCT |
|  | miR396g-DF | AGTCCAAGTACTGCGAGAAGC |
|  | miR396g-DR | GCTCCTTGGACAACGACCA |
|  | miR396h-DF | TCCAAGTACTGCGAGAAGCA |
|  | miR396h-DR | TACGCGTACTCCTTGTGGTC |
|  | miR396d-DF | ACGGTTCCTACTTCGGCAAG |
|  | miR396d-DR | TGTGCTCATCTGCCAATGGT |
| Clone *OsGRFs* genes | GRF4-F： | ATGCCTCCCTGTCTCCGGCG |
|  | GRF4-R： | TCAGTCACCATTAGTTGATC |
|  | GRF6-F： | ATGCTGAGCTCGTCGCCCTC |
|  | GRF6-R： | TCAAGGTAGACGCGGACCGA |
|  | GRF8-F： | ATGCTGAGCTCTTGTGGTGG |
|  | GRF8-R： | TTAGAGAAGTGTTGGGACAA |
| Y2H | GRF4-F | CGCCATATGATGGCGATGCCGTATGCCTC |
|  | GRF4-R | CCGGAATTCTCAGTCACCATTAGTTGATC |
|  | GRF6-F | CGCCATATGATGCTGAGCTCGTCGCCCTC |
|  | GRF6-R | CCGGAATTCTCAAGGTAGACGCGGACCGA |
|  | GRF8-F | CGCCATATGATGCTGAGCTCTTGTGGTGG |
|  | GRF8-R | CCGGAATTCTTAGAGAAGTGTTGGGACAA |
|  | GIF1-F | CGCCATATGATGCAGCAGCAACACCTGATGC |
|  | GIF1-R | CCGGAATTCCTAGCTGCCTTCCTCCTCGG |
|  | GIF2-F | CGCCATATGATGCAGCAGCAGATGGCCAT |
|  | GIF2-R | CCGGAATTCTCAGGACTTCTCCGCTATGTC |
|  | GIF3-F | CGCCATATGATGCAGCAGCAGCCGATGCC |
|  | GIF3-R | CCGGAATTCTTAGGATTGATCGCCGAAGTC |
| BiFC | GRF4-F | AAAAAAGCAGGCTCAGGGGATATCATGGCGATGCCGTATGCCTC |
|  | GRF4-R | GTCTTTGTAGTCCTCGACGATATCGTCACCATTAGTTGATCGAG |
|  | GRF6-F | AAAAAAGCAGGCTCAGGGGATATCATGCTGAGCTCGTCGCCCTC |
|  | GRF6-R | GTCTTTGTAGTCCTCGACGATATCAGGTAGACGCGGACCGATAG |
|  | GRF8-F | AAAAAAGCAGGCTCAGGGGATATCATGCTGAGCTCTTGTGGTGG |
|  | GRF8-R | GTCTTTGTAGTCCTCGACGATATCGAGAAGTGTTGGGACAATAG |
|  | GIF1-F | AAAAAAGCAGGCTCAGGGGATATCATGCAGCAGCAACACCTGATGC |
|  | GIF1-R | GAAAGCTGGGTGCAGGGCGATATCGCTGCCTTCCTCCTCGGTGC |
|  | GIF2-F | AAAAAAGCAGGCTCAGGGGATATCATGCAGCAGCAGATGGCCAT |
|  | GIF2-R | GAAAGCTGGGTGCAGGGCGATATCGGACTTCTCCGCTATGTCACC |
|  | GIF3-F | AAAAAAGCAGGCTCAGGGGATATCATGCAGCAGCAGCCGATGCC |
|  | GIF3-R | GAAAGCTGGGTGCAGGGCGATATCGGATTGATCGCCGAAGTCGCT |
| Detecting GIF1 mutations | GIF1-D-F： | ATCAGGACGCCAATCTCCAG |
|  | GIF1-D-R： | AACAGGAGGGTGCTAGGTAT |
| Construct miR-resistant vector | GRF4-1F | TCGAGCTCGGTACCCGGGGGGCAAGAGAACTATCCGC |
|  | GRF4-1R | GACAGGTTTCCTTGACCGGTTGCGGCCGCGGTGCAT |
|  | GRF4-2F | GTCAAGGAAACCTGTCGAAACGCAGCTGGTCGCCCA |
|  | GRF4-2R | TGCAGGTCGACTCTAGAGTTAGTAGGACACTGTCATCAT |
|  | GRF6-1F | TCGAGCTCGGTACCCGGTAGGCTTCTCGTTGCACACC |
|  | GRF6-1R | GACAGGTTTCCTTGACCGGTTGCGGCCACGGTGCAT |
|  | GRF6-2F | GTCAAGGAAACCTGTCGAATCCAAGACCGCTGCCCCT |
|  | GRF6-2R | TGCAGGTCGACTCTAGAAATACCACAACGTCACGCCT |
|  | GRF8-1F | TCGAGCTCGGTACCCGGGGATGCAAGCCAAGGAAAGGA |
|  | GRF8-1R | GACATGTTTCCTTGACCGATGGCGACCACGGTTTA |
|  | GRF8-2F | GTCAAGGAAACATGTCGAAGGCCGAAAGGCGACACTC |
|  | GRF8-2R | TGCAGGTCGACTCTAGAACTCTTGAAAAGCTGGAGCAGA |
| RACE | GRF4-RACE-R： | GGGTGCCTGAGAATGACGAA |
|  | GRF6-RACE-R： | ATCCATCTCCGACCACGAGT |
|  | GRF8-RACE-R： | GCGGTGCCACCACTATTTAC |
| miR396eGUS experiment | miR396e promoter-F | ACAAGAAGCTTTTAAAGAAATTCCTTGCACA |
|  | miR396e promoter-R | CATGCCATGGCACATCTTTCCCCCCACATCA |
| RT-qPCR | OsAAP1-F | AGCATGGGTGCGATCAAACG |
|  | OsAAP1-R | TTGGTCGTGTCAGATGCCAAGC |
|  | OsAAP3-F | TGCGCGAACAATATCAGGTGCTAC |
|  | OsAAP3-R | AACTCCAACCTCAACGCCAGTC |
|  | OsAAP7-F | CGTCATGCTGCTCTTCTCGTTC |
|  | OsAAP7-R | ACCTTGATGCCACTCAGGTTGG |
|  | OsAAP16-F | TCCCTGCCGTTGTGATCATTGC |
|  | OsAAP16-R | AGAAACAGCACCTCCAGCCATC |
|  | OsUBQ1-F | AACCAGCTGAGGCCCAAGA |
|  | OsUBQ1-R | ACGATTGATTTAACCAGTCCATGA |
|  | OsNIR1-F | CTGCCTCACCAAGGACAG |
|  | OsNIR1-R | TTCCTACTCCTCGTCCTCCT |
|  | OsNIR2-F | GAACGAGGAGTAGGAGCACA |
|  | OsNIR2-R | GGGCTACAAGATCAAACCAA |
|  | OsGOGAT2-F | CCTGTCGAAGGATGATGAAGGTGAAACC |
|  | OsGOGAT2-R | TGCATGGCCCTACTATCTTCGCATCA |
|  | OsGS1.2-F | TGTTTCTCCTCATCCCTGC |
|  | OsGS1.2-R | TCACAGTCCTCGCTTTGC |
| *OsGRF8* , miR396e/f transient expression in *N. benthaminana* leaves | GRF8-XmaI-F | TCCCCCCGGGATGCTGAGCTCTTGTGGTGG |
|  | GRF8-SpeI-R | GGACTAGTGAGAAGTGTTGGGACAATATGAGGATT |
|  | GRF8-SpeI-573-R | GGACTAGTACGGTTTATGTGTCGCTCAC |
|  | GRF8-SpeI-693-R | GGACTAGTCTTCTGCCGAGCCACAGTG |
|  | mir396e-XmaI-F | TCCCCCCGGGTATATCCCCCCGCCTCAGTG |
|  | mir396e-PstI-R | AACTGCAGAAAATTCTCCGCCGCAACG |
|  | mir396f-XmaI-F | TCCCCCCGGGATATGTATGTACGTACGGATCGATCAAAAG |
|  | mir396f-PstI-R | AACTGCAGTTTACCTGCTAGATCGAATTGCACG |
